# Variation in Concentration and Sources of Black Carbon in a Megacity of China During the COVID‐19 Pandemic

**DOI:** 10.1029/2020GL090444

**Published:** 2020-11-28

**Authors:** Liang Xu, Jian Zhang, Xin Sun, Shengchen Xu, Meng Shan, Qi Yuan, Lei Liu, Zhenhong Du, Dantong Liu, Da Xu, Congbo Song, Bowen Liu, Gongda Lu, Zongbo Shi, Weijun Li

**Affiliations:** ^1^ Key Laboratory of Geoscience Big Data and Deep Resource of Zhejiang Province, Department of Atmospheric Sciences, School of Earth Sciences Zhejiang University Hangzhou China; ^2^ Zhejiang Ecological and Environmental Monitoring Center Hangzhou China; ^3^ Zhejiang Linan Atmospheric Background National Observation and Research Station Hangzhou China; ^4^ School of Geography, Earth and Environmental Sciences University of Birmingham Birmingham UK; ^5^ Department of Economics University of Birmingham Birmingham UK

**Keywords:** black carbon, COVID‐19 lockdown, source

## Abstract

Black carbon (BC) not only warms the atmosphere but also affects human health. The nationwide lockdown due to the Coronavirus Disease 2019 (COVID‐19) pandemic led to a major reduction in human activity during the past 30 years. Here, the concentration of BC in the urban, urban‐industry, suburb, and rural areas of a megacity Hangzhou were monitored using a multiwavelength Aethalometer to estimate the impact of the COVID‐19 lockdown on BC emissions. The citywide BC decreased by 44% from 2.30 to 1.29 μg/m^3^ following the COVID‐19 lockdown period. The source apportionment based on the Aethalometer model shows that vehicle emission reduction responded to BC decline in the urban area and biomass burning in rural areas around the megacity had a regional contribution of BC. We highlight that the emission controls of vehicles in urban areas and biomass burning in rural areas should be more efficient in reducing BC in the megacity Hangzhou.

## Key Points

- BC concentrations during the COVID‐lockdown were reduced by 44% in a megacity of China
- BC from fossil fuels has the largest reduction in urban area during the COVID‐lockdown
- Vehicles and industrial activities are the major contributors of BC in the megacity, followed by and biomass burning

## 1 Introduction

Rapid industrialization and urbanization over the past few decades has led to severe air pollution across China, which poses adverse effects on public health (Lelieveld et al., 2020; Zhang, Wang, Guo, et al., 2015). To address issues of air pollution, the State Council of China promulgated the Air Pollution Prevention and Control Action Plan in September 2013 (State Council of the People's Republic of China, 2013). A recent study has shown that air quality in China has improved significantly. In particular, from 2013 to 2017, the decline in the annual fine particulate matter (PM_2.5_) concentrations was 39%, 34%, and 26% in Beijing‐Tianjin‐Hebei, the Yangtze River Delta, and the Pearl River Delta, respectively (Wang et al., 2018). However, extreme haze events occasionally occur, especially in winter and spring, due to multiple factors, such as unfavorable meteorology, regional transport, and heterogeneous reactions (Zhang, Wang, Wang, et al., 2015). Therefore, more efforts are required for the further improvement in the air quality.

Controlling anthropogenic emissions is an effective way to mitigate the air pollution level. Various emission reduction campaigns have been employed to improve the local air quality during several major events, including the 2008 Beijing Olympic (Okuda et al., 2011), the 2014 Asia‐Pacific Economic Cooperation (APEC) meeting (Zhang, Li, et al., 2018), the 2015 China Victory Day Parade (Zhao et al., 2017), and the 2016 G20 summit (Li et al., 2018). During these special events, some factories with high emissions were temporarily closed, construction activities were stopped, and the number of vehicles was restricted (Sun et al., 2016). These special emission reduction periods are valuable for both the government and scientists to assess the effectiveness of the control measures on air quality improvement. However, these previous campaigns were only regional with a few control days, which might have influenced their significance.

As the first country reporting and fighting Coronavirus Disease 2019 (COVID‐19), China has implemented a series of effective measures to prevent the spread of COVID‐19, including stay‐at‐home orders, travel bans, shutting down nonessential commercial activities, and a national lockdown (Tian et al., 2020). These nationwide restrictions have led to the lowest human activity in modern China in the past 30 years, resulting in the lowest anthropogenic pollutant emissions and the mitigation of the air pollution level (Figure S1). The unprecedented COVID‐19 lockdown could provide us with a comprehensive understanding of the sources of air pollutants and our current emission control strategies. Revealing the changes in pollutant emissions during the COVID‐19 is important for future emission control strategies to improve air quality in China.

Black carbon (BC) is a primary aerosol emitted from the incomplete combustion of fossil fuels and biomass. BC has been considered the second greatest contributor to global warming, just after CO_2_ (Bond et al., 2013). BC coated with other aerosol materials could further alter light absorption (Cappa et al., 2019). Studies on BC are a continuous hot topic in atmospheric sciences and climate change (Liu, Whitehead, et al., 2017). The main sources of BC include (1) on‐road and off‐road diesel engines; (2) industrial coal combustion; (3) residential solid fuels (wood, crop residue, and coal); and (4) open burning of biomass (Chen et al., 2017; Liu, Kong, et al., 2017; Wang et al., 2017; Zhang, Yuan, et al., 2018). However, it is difficult to know the BC source apportionment in cities because BC does not have specific chemical tracers among the different sources (Bond et al., 2013). Here, the COVID‐19 period provides a unique opportunity to understand the BC sources and concentrations, which can be helpful for policy makers to reduce BC at the city level.

Many studies have reported the variations in air pollutants due to the COVID‐19 pandemic. They revealed the variations of SO_2_, NO_x_, and PM_2.5_ concentration from a regional perspective based on data from satellites (Bauwens et al., 2020; Zhang et al., 2020), ground‐based national environmental monitoring centers (Cui et al., 2020; Huang et al., 2020; Yuan et al., 2021), or model simulations (Huang et al., 2020; Wang et al., 2020). However, this is the first study that focuses on the BC variations from ground measurements during the COVID‐19 lockdown at the megacity level. In this study, we present the measurements of BC during the COVID‐19 outbreak using a multi‐wavelength Aethalometer in the urban, urban‐industry, suburban, and rural areas of Hangzhou city, located in the southern part of the Yangtze River Delta (YRD) in East China. We investigated and compared the variability and diurnal changes in the BC concentrations before, during, and after the COVID‐19 lockdown. Moreover, the absorption Ångström exponents (AAEs) of BC were used to estimate the contribution from different BC emission sources.

## 2 Experiments

### 2.1 Sampling areas

**Figure 1:**
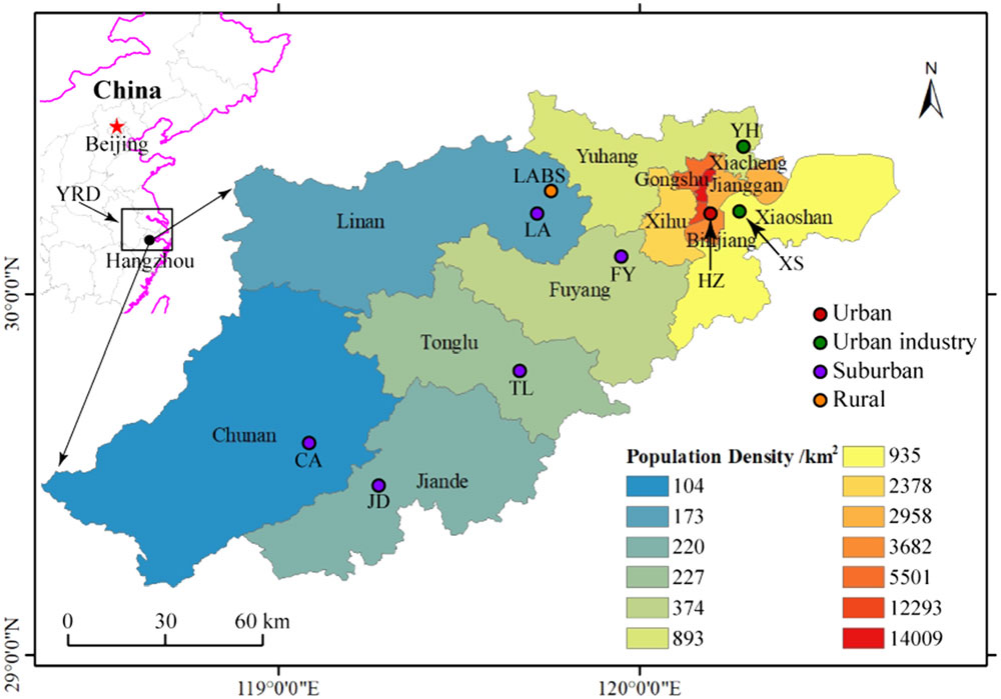
The location of observation sites and the population density of Hangzhou City.

The BC observations presented in this study were carried out from 1 January to 31 March 2020 at nine observation sites in Hangzhou (Figure 1). Hangzhou, as the capital of Zhejiang Province, is located in the northern part of Zhejiang. Hangzhou is also the second largest city in the Yangtze River Delta (YRD), one of the most developed areas in China. Based on the population density, the number and output of industries, and the number of private vehicles from different sites (Figure 1 and Table S1), we classified these nine sites into four categories: urban, urban‐industry, suburban, and rural.

#### 2.1.1 Urban: Hangzhou (HZ)

The HZ site is located in an urban area, which has the highest population density and the highest private vehicle number. The site is surrounded by residential and commercial buildings, without obvious effects from industrial activities. The main BC sources at this site are from traffic and residential activities.

#### 2.1.2 Urban‐industry: Yuhang (YH) and Xiaoshan (XS)

These two urban sites belong to two districts with some heavy industrial enterprises (Figure S2). The population density and private vehicle number (Figure 1 and Table S1) indicate that traffic and residential activities could contribute to BC emissions. Therefore, both vehicle and industrial activities are the major sources of BC at urban‐industrial sites.

#### 2.1.3 Suburban: Fuyang (FY), Lin'an (LA), Tonglu (TL), Jiande (JD), and Chun'an (CA)

These five sites are located in districts and counties under the jurisdiction of Hangzhou. The population density, private vehicle number, and industrial enterprise number of these areas are much lower than those of the urban and urban industry sites.

#### 2.1.4 Rural: Lin'an Background Site (LABS)

The LABS site is on the top of a mountain at an altitude of 138 m, which is a regional background site for the YRD region. The site is surrounded by forests and small villages. Some villagers in rural areas usually use wood or bamboo for cooking.

### 2.2 BC observation

BC mass concentrations are measured at a 5‐min resolution by an Aethalometer AE‐31 (Magee Scientific, USA) with a flow rate of 5 L min^−1^. The light attenuation due to the deposition of aerosol particles on the filter tape is continuously measured at seven wavelengths (370, 470, 520, 590, 660, 880, and 950 nm). The light attenuation is linearly proportional to the amount of BC in the filter deposit. Detailed information regarding the principle and operation of the Aethalometer can be found in Hansen (2005). It should be noted that an empirical value of the correction factor C = 2.14 (factor to compensate for multiple scattering of the fiber filter) was applied in this study as proposed by Weingartner et al. (2003) and Sandradewi, Prévôt, Weingartner, et al. (2008).

Assuming that biomass burning and fossil fuel emissions only contribute to BC absorption (Equation 1), the AAEs (Equation 2) can be used to estimate the contribution from these two sources based on the Aethalometer model (Sandradewi, Prévôt, Szidat, et al., 2008; Zotter et al., 2017). Pure BC absorbs light over the entire visible wavelength range with a weak spectral dependence (AAE ~ 1). BC from biomass burning contains additional light‐absorbing organic materials that strongly enhance the absorption in the shorter wavelength range (AAE > 1), while fossil fuel‐related BC contains minor other light‐absorbing compounds (AAE ~ 1) (Sandradewi, Prévôt, Szidat, et al., 2008).

(1) $$b_{\mathrm{abs}}\left(\lambda \right)=b_{\mathrm{abs},\mathrm{ff}}\left(\lambda \right)+b_{\mathrm{abs},\mathrm{bb}}\left(\lambda \right)$$

(2) $$\mathrm{AAE}\left(\lambda_{1},\lambda_{2}\right)=-\frac{\ln\left(\frac{b_{\mathrm{abs}}\left(\lambda_{1}\right)}{b_{\mathrm{abs}}\left(\lambda_{2}\right)}\right)}{\ln\left(\frac{\lambda_{1}}{\lambda_{2}}\right)}$$

where b_abs,ff_ (λ) and b_abs,bb_ (λ) are the absorption coefficients apportioned to fossil fuel combustion and biomass burning, respectively; and b_abs_(λ_1_) and b_abs_ (λ_2_) are the absorption coefficients at λ_1_ and λ_2_, respectively.

Using the measured b_abs_ values at two different wavelengths, the source apportionment of BC from fossil fuel (BC_ff_) and biomass burning (BC_bb_) can be derived using the following equations:

(3) $$\frac{b_{\mathrm{abs},\mathrm{ff}}\left(\lambda_{1}\right)}{b_{\mathrm{abs},\mathrm{ff}}\left(\lambda_{2}\right)}={\left(\frac{\lambda_{1}}{\lambda_{2}}\right)}^{-{\mathrm{AAE}}_{\mathrm{ff}}}$$

(4) $$\frac{b_{\mathrm{abs},\mathrm{bb}}\left(\lambda_{1}\right)}{b_{\mathrm{abs},\mathrm{bb}}\left(\lambda_{2}\right)}={\left(\frac{\lambda_{1}}{\lambda_{2}}\right)}^{-{\mathrm{AAE}}_{\mathrm{bb}}}$$

(5) $$b_{\mathrm{abs},\mathrm{bb}}\left(\lambda_{2}\right)=\frac{b_{\mathrm{abs}}\left(\lambda_{1}\right)-b_{\mathrm{abs}}\left(\lambda_{2}\right)\cdot {\left(\frac{\lambda_{1}}{\lambda_{2}}\right)}^{-{\mathrm{AAE}}_{\mathrm{ff}}}}{{\left(\frac{\lambda_{1}}{\lambda_{2}}\right)}^{-{\mathrm{AAE}}_{\mathrm{bb}}}-{\left(\frac{\lambda_{1}}{\lambda_{2}}\right)}^{-{\mathrm{AAE}}_{\mathrm{ff}}}}$$

(6) $$b_{\mathrm{abs},\mathrm{ff}}\left(\lambda_{2}\right)=\frac{b_{\mathrm{abs}}\left(\lambda_{1}\right)-b_{\mathrm{abs}}\left(\lambda_{2}\right)\cdot {\left(\frac{\lambda_{1}}{\lambda_{2}}\right)}^{-{\mathrm{AAE}}_{\mathrm{bb}}}}{{\left(\frac{\lambda_{1}}{\lambda_{2}}\right)}^{-{\mathrm{AAE}}_{\mathrm{ff}}}-{\left(\frac{\lambda_{1}}{\lambda_{2}}\right)}^{-{\mathrm{AAE}}_{\mathrm{bb}}}}$$

where AAE_ff_ and AAE_bb_ are the AAE values of fossil fuel BC and biomass burning BC, respectively. Here, the AAE values used to estimate the fossil fuel and biomass burning contributions are AAE_ff_ = 1.0 and AAE_bb_ = 2.0 according to previous literatures (Sandradewi, Prévôt, Szidat, et al., 2008; Zheng et al., 2019). The AAE selection and uncertainty (40%) for the BC_bb_ and BC_ff_ was discussed in the supporting information.

In this study, we chose BC light absorption at 470 and 950 nm to calculate the AAE values and source apportionment (Becerril‐Valle et al., 2017; Harrison et al., 2013). The absorption at 370 nm was excluded to minimize interference because light‐absorbing secondary organic aerosol, other absorbing non‐BC combustion particles, and atmospheric processing affect lower wavelengths more than higher ones (Zotter et al., 2017). The Aethalometer data recorded during the rainy periods were excluded from the follow‐up BC comparison due to the wet removal of precipitation.

### 2.3 The Demarcation of the Time Periods

**Figure 2:**
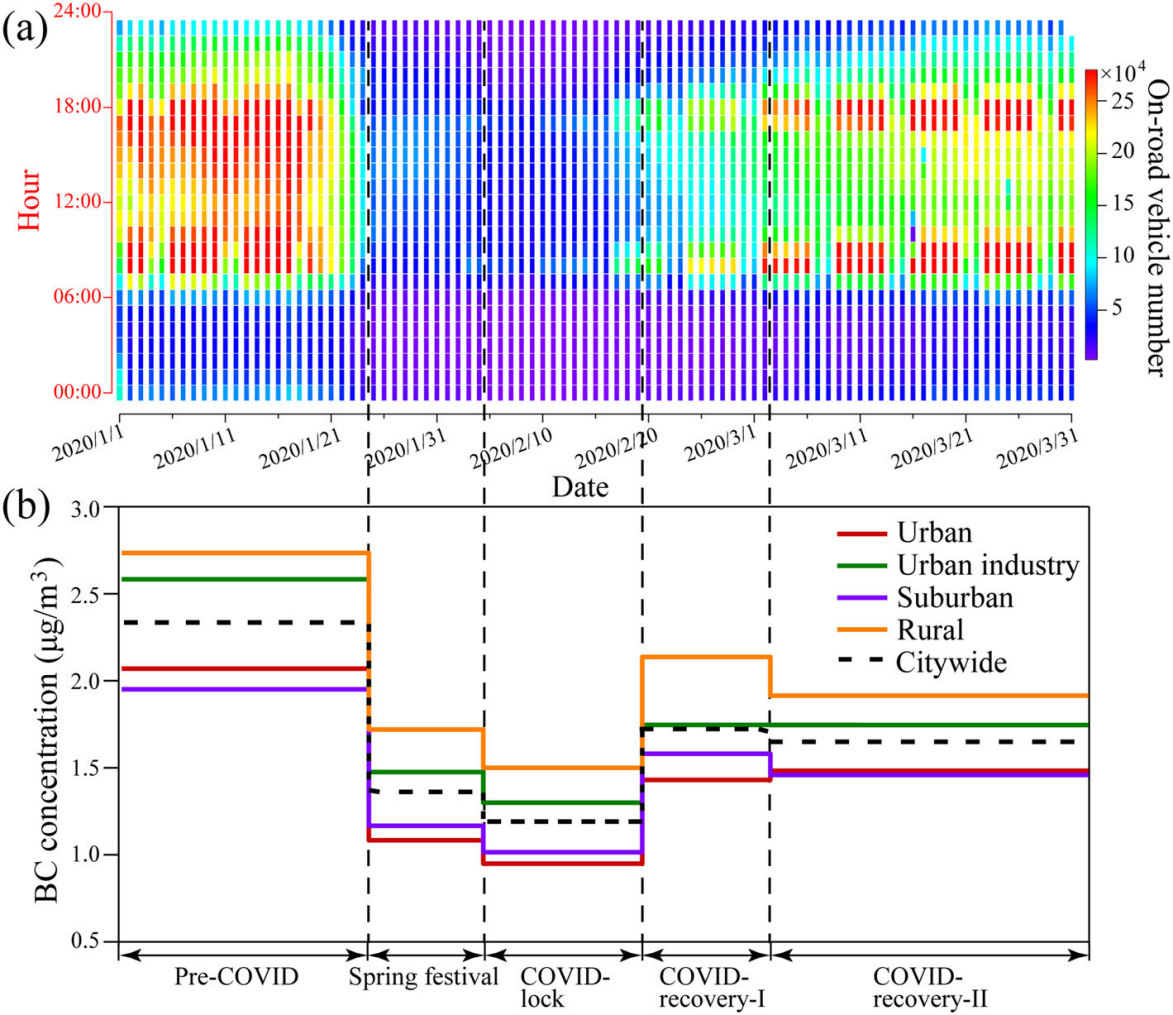
(a) The hourly averaged vehicle number running on the roads showing the variation in traffic in downtown Hangzhou. Data source: Hangzhou Traffic Police Division. The figure was generated using Igor Probased computer programs developed by (Wu et al. (2018)). (b) The mean deweathered BC concentration in different areas during the five stages. The detailed BC concentration is presented in Table S3 in the supporting information.

Figure S3 shows the timeline of the COVID‐19 outbreak and regulations issued by the government for epidemic control. The novel coronavirus was identified and reported in Wuhan City on 31 December 2019. WHO confirmed human‐to‐human transmission of the virus on 21 January 2020 (WHO, 2020). Two days later on 23 January, Wuhan City, the epicenter of COVID‐19 was locked down. On the same day, the first‐level emergency response for epidemic prevention and control was issued by Zhejiang Province. On 4 February, strict epidemic prevention and control measures were issued by the Hangzhou municipal government. In the middle of February, factories and public spaces started to reopen as the COVID‐19 cases decreased in China. Zhejiang Province downgraded the emergency response levels to the second level on 2 March. Figure 2a shows the hourly averaged vehicle number running on the roads calculated from the microwave detector data set on the roads in downtown Hangzhou. The traffic flow data in this study were provided by Hangzhou Traffic Police Division. A sharp decrease (84%) in traffic was observed during the epidemic control period (average values, 165,000 for the pre‐COVID, 25,800 for the COVID‐19 lockdown).

Based on the timeline in Figure S3 and traffic in Figure 2a, we define five stages: pre‐COVID (1–23 January), spring festival (24 January to 4 February), COVID‐19 lockdown (5–19 February), COVID‐recovery‐I (20 February to 2 March), and COVID‐recovery‐II (2–31 March). The pre‐COVID stage is the reference stage without the influence of COVID‐19. In the spring festival stage, the public has realized the seriousness of COVID‐19, but strict lockdown policy was not implemented yet. There was a certain amount of anthropogenic BC emission from vehicles and industry. Meanwhile, due to interference from fireworks and firecrackers during the Spring Festival holiday, we separate this stage from the COVID‐19 lockdown stage as a spring festival. The COVID‐19 lockdown stage is the stage with the strictest epidemic control measures mentioned above. Both the COVID‐recovery‐I and ‐II stages are the restoration stages for working and living after the control of the spread of COVID‐19. Stage II is the period with the highest rate of economic restoration.

## 3 Results and Discussion

### 3.1 Diurnal Variation in BC During Different Periods

**Figure 3:**
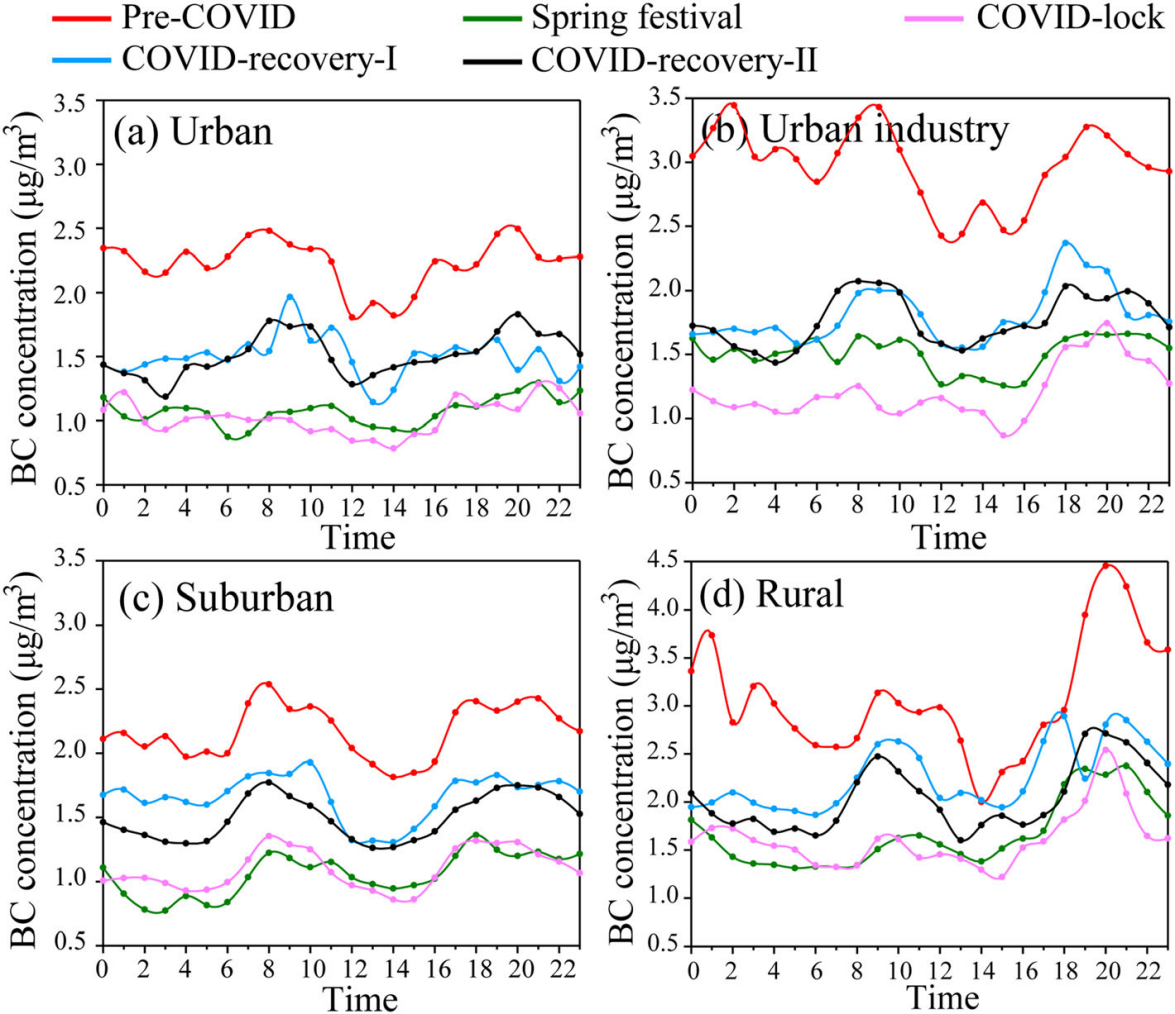
Mean diurnal variations in BC (without deweathering) in the four areas on weekdays. (a) Urban, (b) Urban industry, (c) Suburban, and (d) Rural.

Figure 3 illustrates the mean diurnal BC concentrations of Hangzhou in four areas on weekdays. The bimodal distribution of the BC concentration is observed in each area during the pre‐COVID and COVID recovery‐I and ‐II stages, with the morning peak at 7:00–9:00 and the evening peak at 19:00–21:00. The morning BC peak is close to the evening peak and 1.5 times higher than the noon BC concentration in the four areas. However, the morning peak during the COVID‐19 lockdown disappears in the urban and urban‐industry areas, but a weak evening peak still exists. In the suburban and rural areas, similar morning and evening BC peaks were still observed. Here, we assume a same daily variation in the planetary boundary layer (PBL) height because of the similar meteorological parameters during the pre‐COVID and COVID‐19 lockdown (Figure S4). The household cooking emissions from wood and bamboo burning in the villages likely caused the weak bimodal diurnal variation in the suburban and rural areas during the COVID‐19 lockdown. Considering the urban morning BC concentration during the COVID‐19 lockdown as a reference, we estimate that BC from the villages contribute 0.27 and 0.43 μg/m^3^ in the morning peak in the suburb and rural areas during the COVID‐19 lockdown, respectively. Moreover, the decline of BC peak concentration (rush hours) in the urban and urban industrial areas during COVID‐19 lockdown could be attributed to traffic reduction. We compared the BC peaks during the pre‐COVID and COVID‐19 lockdown and then estimated that the traffic reduction contributed to 1.33 μg/m^3^ BC peak decline in the urban area, 1.84 μg/m^3^ in the urban industrial area, accounting for 55% and 57% of the total BC, respectively (Figure 3). Therefore, comparisons of the diurnal variations in BC in different areas can roughly reflect local source contributions.

### 3.2 Reduction in the BC Concentration

Changes of weather conditions could cause variations in the concentration of air pollutants even with the constant emissions. To estimate real changes in emissions, it is important to decouple the effects of weather from emission‐related changes (Grange & Carslaw, 2019). Here, we applied a machine‐learning based random forest (RF) algorithm to decouple the effects of meteorological conditions (Grange et al., 2018; Grange & Carslaw, 2019; Vu et al., 2019). The RF model was built using the “rmweather” R package by Grange et al. (2018), 70% of the original data were randomly selected to train the model, which was then evaluated with the rest (30%) of the data set. The detailed information about this algorithm and model performance is introduced in the supporting information (Table S5). The BC concentrations compared in the follow‐up sections are all based on the deweathered data.

The original observed BC concentrations were 2.33 μg/m^3^ during the pre‐COVID, 1.36 μg/m^3^ during the spring festival, 1.20 μg/m^3^ during the COVID‐lock, and 1.70 μg/m^3^ during the COVID‐recovery period (Figure S5). The deweathered average BC concentrations were 2.30 μg/m^3^ during the pre‐COVID stage, 1.37 μg/m^3^ during the spring festival, 1.29 μg/m^3^ during the COVID‐19 lockdown period, and 1.45 μg/m^3^ during the COVID recovery period (Figure 2b). Therefore, we can conclude that the meteorological conditions led to little difference between the deweathered and observed BC concentrations during the pre‐COVID, spring festival, and COVID‐lockdown stage.

The deweathered result shows that the BC reduced by 44% from the pre‐COVID stage to the COVID‐19 lockdown stage, suggesting the significant mitigation of BC from the control measures. Figure 2a shows vehicles running on the road have reached the lowest point during the COVID‐19 lockdown, suggesting minimum BC from the various vehicles in the city. Therefore, a majority of BC (1.29 μg/m^3^) during the COVID‐19 lockdown should be considered as the regional contribution from heavy industries and rural biomass burning.

Table S2 compares BC during the previous emission control events in the megacities of China. We found that the BC concentration in Hangzhou (1.29 μg/m^3^) during COVID‐1ock was much lower than that (3.2 μg/m^3^) in Beijing during the 2008 Olympics (Okuda et al., 2011) and the concentration (2.5 μg/m^3^) during the 2014 APEC (Zhang, Li, et al., 2018) but was close to that (1.20 μg/m^3^) during the 2015 China Victory Day parade (Zhao et al., 2017). The 44% BC reduction from the COVID‐19 lockdown to the pre‐COVID period is twice as much as the control periods during the 2016 G20 summit in Hangzhou (Li et al., 2018). These comparisons show that the BC during the COVID‐19 lockdown maintained the lowest concentration in the megacity.

Figure 2b also shows similar patterns of the BC concentrations in different areas in Hangzhou. Compared with the deweathered BC concentrations during the pre‐COVID, BC during the COVID‐19 lockdown decreases by approximately 0.93, 1.27, 0.79, and 1.02 μg/m^3^ in urban, urban industry, suburban, and rural areas, respectively, and their corresponding decreasing percentages are 47%, 49%, 41%, and 38%, respectively (Table S3).

We compared the on‐road‐vehicle number before, during, and after the COVID‐19 lockdown. Figure 2a shows that the traffic flow decrease ratio was 84% in the urban area. Gasoline and diesel vehicles have different BC emission characteristics, BC emissions from heavy‐duty diesel vehicles can be 50 times higher than light‐duty gasoline vehicles (Dallmann et al., 2012, 2013). Therefore, different reduction proportion of gasoline and diesel vehicles may affect BC emission in the city. Here we randomly chose five roads in the urban area to obtain the number of car and truck running on the roads in daily resolution (car: gasoline engine, truck: diesel engine, Figure S6). The results show that gasoline and diesel vehicles experience similar reduction proportion between pre‐COVID and COVID‐lock period (82% and 83%, respectively). Urban areas, situated in residential and commercial districts, can consider traffic emissions as the major contributor to particulate matters (Wu et al., 2016). Therefore, the 0.93 μg/m^3^ BC decline in the urban area can be attributed to traffic flow decreasing. As a result, we can estimate that traffic emissions only contributed to 0.18 μg/m^3^ BC in total BC at 1.06 μg/m^3^ in the urban area during the COVID‐19 lockdown. The urban industrial area is under the dual influence of traffic and industrial activities (Table S1). If the BC decline in the urban area is considered to be the reference of traffic, then we can estimate that the BC decline from industrial activities is about 0.34 μg/m^3^ in the urban‐industrial area.

Interestingly, Figure 2b shows that the highest BC concentrations in Hangzhou continuously occurred in the rural area in all five stages. It is likely considerate that biomass burning for household cooking is an important source of BC in the rural areas. If the BC in urban areas is considered to be the regional background during the COVID‐19 lockdown, then we estimate concentrations of 0.07 and 0.59 μg/m^3^ from biomass burning in the suburban and rural areas, respectively. Based on the different emission characteristics among the rural, urban, and urban industrial areas, we can roughly estimate the contribution from traffic and industrial activities for BC reduction during the COVID‐19 lockdown.

### 3.3 Contribution From Different Sources for BC Mitigation

The Aethalometer model can apply the AAE to evaluate the BC contributions of fossil fuels and biomass (Sandradewi, Prévôt, Szidat, et al., 2008; Zotter et al., 2017). Previous studies have reported AAE values near 1 that are related to BC from fossil fuels and much higher AAE values from biomass burning (Becerril‐Valle et al., 2017; Sandradewi, Prévôt, Szidat, et al., 2008; Zotter et al., 2017). In this study, the AAE values in different areas increased from pre‐COVID and reached their largest value (1.17, 1.12, 1.21, and 1.17) during the COVID‐19 lockdown, suggesting that BC from fossil fuels sharply decreased in Hangzhou during the COVID‐19 lockdown (Figure S7). In particular, the AAEs in the suburban and rural areas are much higher than those in the urban and urban industry areas. The result is consistent with the distribution of BC concentrations in different areas, suggesting that biomass burning in the suburban and rural areas continuously contributed to BC during the COVID‐19 lockdown in Hangzhou.

To apportion BC sources, we conducted further calculations for each weekday based on Equations 5 and 6 from the Aethalometer model (Zotter et al., 2017). Harrison et al. (2013) reminded that estimates of woodsmoke from Aethalometer data were sensitive to choice of AAE. Here, we applied the optimal combination of AAE_ff_ = 1.0 and AAE_bb_ = 2.0 to quantify BC source apportionment (Sandradewi, Prévôt, Szidat, et al., 2008; Zheng et al., 2019). Figure S8 presents the relative contribution of biomass burning to the total BC (*f*_bb_) on weekdays. The *f*_bb_ values range from 5–9% in the urban, urban industry, and suburban areas during the pre‐COVID period but increase up to ~20% during the spring festival and COVID‐19 lockdown. This result suggests that the increasing BC fraction is from biomass burning in the air. As expected, *f*_bb_ returns to lower values at ~10% in the COVID‐recovery‐I and ‐II periods.

**Figure 4:**
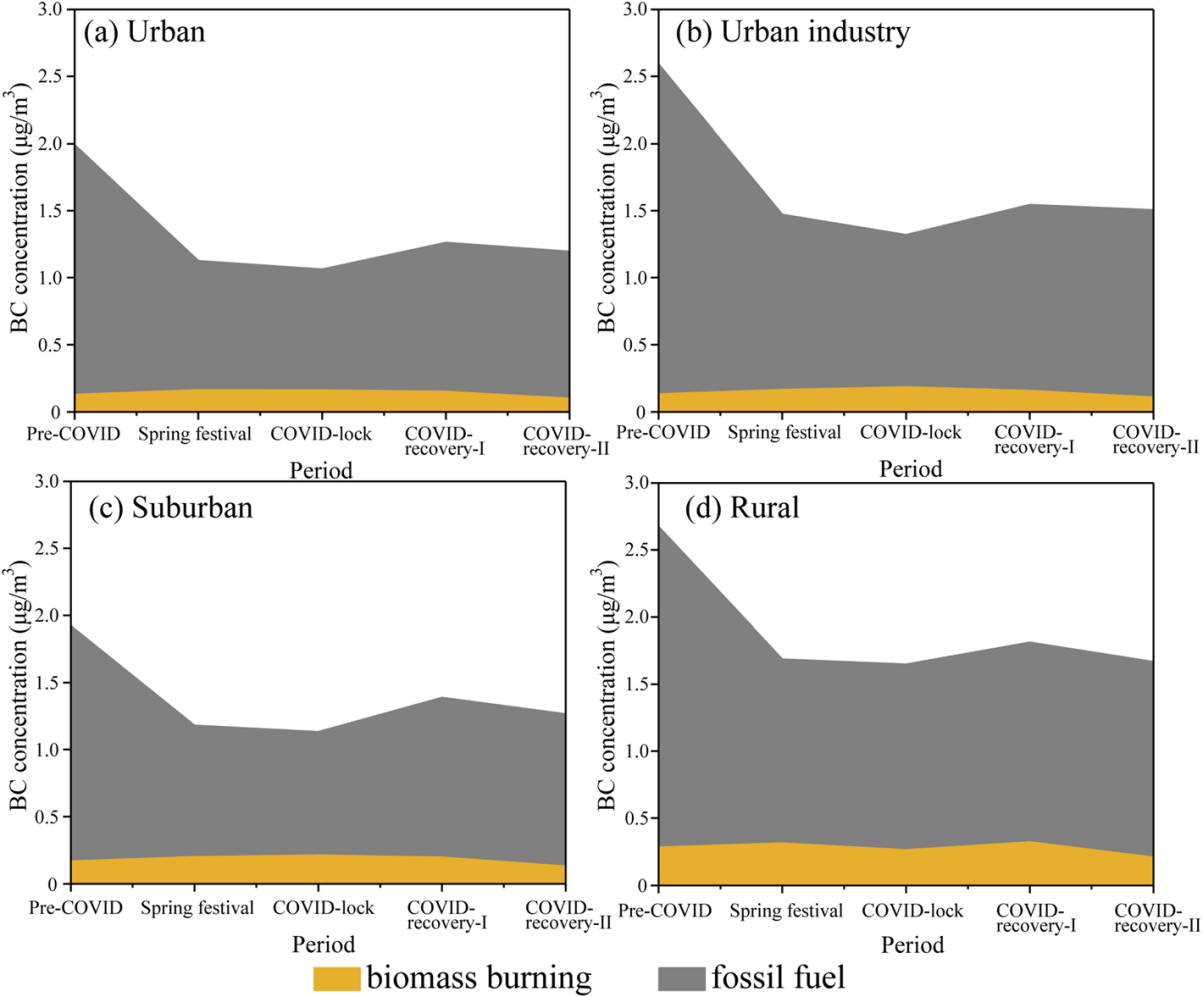
Average variation in the BC source apportionment from fossil fuel (gray area) and biomass burning (orange area) for the four areas on weekdays (deweathered data). (a) Urban, (b) Urban industry, (c) Suburban, and (d) Rural. The detailed data are presented in Table S4.

Based on *f*_bb_ and total BC concentrations, we can obtain the BC concentration from fossil fuel (BC_ff_) and biomass burning (BC_bb_) on weekdays (Figures 4 and S9). The BC_ff_ in the urban area decreased from 1.86 μg/m^3^ in the pre‐COVID period to 0.90 μg/m^3^ during the COVID‐19 lockdown. This 0.96 μg/m^3^ BC_ff_ decline in the urban area can be roughly attributed to vehicle emissions (Table S1 and Figure 2a). The value is consistent with the 0.93 μg/m^3^ BC decline in the urban area, as estimated in section 3.2. In the urban industry area, BC_ff_ decreased from 2.46 μg/m^3^ during the pre‐COVID period to 1.13 μg/m^3^ during the COVID‐19 lockdown. If we consider a 0.96 μg/m^3^ BC reduction in the urban area as a reference for vehicle emissions, then the BC decline from industrial activities is estimated to be 0.37 μg/m^3^. The result is slightly higher than the BC decline of 0.34 μg/m^3^ from the industry area estimated in section 3.2. Moreover, we further calculated vehicle emissions in the suburban and rural areas and found a 48% and 42% BC_ff_ decline during the COVID‐19 lockdown, respectively (Table S4).

Figure 4d shows that the BC_bb_ value in the rural area demonstrated the highest level during our observation from 0.21 to 0.32 μg/m^3^. The result is reasonable because biomass fuels (e.g., wood and bamboo) are frequently used as energy sources for cooking in villages. It should be noted that lower BC_bb_ concentrations and small fluctuations (0.13–0.21 μg/m^3^) occurred in the urban, urban industry, and suburban areas during the first three stages (Figure S9). The result suggests that biomass burning in rural areas has a regional contribution to BC in Hangzhou.

Based on the data analysis, we conclude that vehicles and industrial activities are the major sources of BC in Hangzhou, but BC from biomass burning in the suburban and rural areas should be given more attention. The result is consistent with the previous study that applied radiocarbon Δ^14^C and stable carbon δ^13^C to estimate the BC source apportionments in 2013: 24% for coal (industrial sector), 44% for liquid fossils (transportation sector) and 32% for biomass (residential sector) in the YRD (Fang et al., 2018).

## 4 Conclusions and Atmospheric Implications

In this study, the BC reduction due to restricted anthropogenic activities during the COVID‐19 outbreak was investigated in Hangzhou from 1 January to 31 March 2020. The anthropogenic emission decrease significantly contributed to the decrease in the BC concentration. The citywide BC concentrations during the COVID‐19 lockdown decreased by 44% (2.30 to 1.29 μg/m^3^) compared with those during the pre‐COVID period. The BC during the COVID‐19 lockdown decreased by ~0.93, 1.27, 0.79, and 1.02 μg/m^3^ in urban, urban industry, suburban, and rural areas, respectively, and their corresponding decreasing percentages are 47%, 49%, 41%, and 38%, respectively. The BC decline from different sources was assessed based on the emission characteristics in urban and urban industrial areas. We found a 0.93 μg/m^3^ BC decline in the urban and urban industry areas due to a reduction in vehicle emissions and a 0.34 μg/m^3^ BC decline due to a reduction in industrial activities in the urban industrial area.

The BC reduction and source assessment during the COVID‐19 lockdown in Hangzhou provides us with a clear picture of how to reduce BC in the megacities of China in the future. The BC concentration at 2.30 μg/m^3^ during the pre‐COVID period in Hangzhou is relatively lower than that in other areas of China (e.g., 4.58 μg/m^3^ in Beijing, January 2016, Liu et al., 2018). However, the significant BC decline due to the unprecedented COVID‐19 lockdown reminds us that BC mitigation with 0.93 μg/m^3^ (44% in percentage) can be achieved in the future. Vehicle emission reduction is the primary contributor to BC decline in the urban area of Hangzhou. Therefore, the continuous growth of new energy vehicles might mitigate the BC concentration. Moreover, the driving restriction policy during weekdays is still an effective way to reduce vehicle emissions in Hangzhou. We also found that the regional contribution of BC from biomass burning in rural villages is still a considerable source of BC in Hangzhou, which requires more control measures in the future.

A meta‐analysis study indicates that a 1 μg/m^3^ BC increase is associated with a 1.45% increase in all‐cause mortality and a 1.77% increase in cardiovascular mortality (Janssen et al., 2011). BC in the megacities of China could depress the development of PBL and consequently enhance the occurrences of extreme haze pollution events (Ding et al., 2016). A study based on a climate model shows that BC can lead to the enhancement of precipitation in East China (Jiang et al., 2013). The BC reduction during the COVID‐19 lockdown has mitigated BC in China as a whole, which in the long run could benefit the population health and regional climate.

## Conflict of Interest

The authors declare no competing financial interests.

## Supporting information

Supporting Information S1

## Data Availability

The data used in this study are publicly available at http://figshare.com (https://doi.org/10.6084/m9.figshare.12751547.v1).

## References

[grl61575-bib-0001] Bauwens, M. , Compernolle, S. , Stavrakou, T. , Müller, J.‐F. , van Gent, J. , Eskes, H. , Levelt, P. F. , Van Der, A. R. , Veefkind, J. P. , Vlietinck, J. , & Yu, H. (2020). Impact of coronavirus outbreak on NO_2_ pollution assessed using TROPOMI and OMI observations. Geophysical Research Letters, 47, e2020GL087978 10.1029/2020gl087978 PMC7261997 32836515

[grl61575-bib-0002] Becerril‐Valle, M. , Coz, E. , Prévôt, A. S. H. , Močnik, G. , Pandis, S. N. , Sánchez de la Campa, A. M. , Alastuey, A. , Díaz, E. , Pérez, R. M. , & Artíñano, B. (2017). Characterization of atmospheric black carbon and co‐pollutants in urban and rural areas of Spain. Atmospheric Environment, 169, 36–53. 10.1016/j.atmosenv.2017.09.014

[grl61575-bib-0003] Bond, T. C. , Doherty, S. J. , Fahey, D. W. , Forster, P. M. , Berntsen, T. , DeAngelo, B. J. , Flanner, M. G. , Ghan, S. , Kärcher, B. , Koch, D. , Kinne, S. , Kondo, Y. , Quinn, P. K. , Sarofim, M. C. , Schultz, M. G. , Schulz, M. , Venkataraman, C. , Zhang, H. , Zhang, S. , Bellouin, N. , Guttikunda, S. K. , Hopke, P. K. , Jacobson, M. Z. , Kaiser, J. W. , Klimont, Z. , Lohmann, U. , Schwarz, J. P. , Shindell, D. , Storelvmo, T. , Warren, S. G. , & Zender, C. S. (2013). Bounding the role of black carbon in the climate system: A scientific assessment. Journal of Geophysical Research: Atmospheres, 118, 5380–5552. 10.1002/jgrd.50171

[grl61575-bib-0005] Cappa, C. D. , Zhang, X. , Russell, L. M. , Collier, S. , Lee, A. K. Y. , Chen, C.‐L. , Betha, R. , Chen, S. , Liu, J. , Price, D. J. , Sanchez, K. J. , McMeeking, G. R. , Williams, L. R. , Onasch, T. B. , Worsnop, D. R. , Abbatt, J. , & Zhang, Q. (2019). Light absorption by ambient black and brown carbon and its dependence on black carbon coating state for two California, USA, cities in winter and summer. Journal of Geophysical Research: Atmospheres, 124, 1550–1577. 10.1029/2018JD029501

[grl61575-bib-0006] Chen, J. , Li, C. , Ristovski, Z. , Milic, A. , Gu, Y. , Islam, M. S. , Wang, S. , Hao, J. , Zhang, H. , He, C. , Guo, H. , Fu, H. , Miljevic, B. , Morawska, L. , Thai, P. , Lam, Y. F. , Pereira, G. , Ding, A. , Huang, X. , & Dumka, U. C. (2017). A review of biomass burning: Emissions and impacts on air quality, health and climate in China. Science of the Total Environment, 579, 1000–1034. 10.1016/j.scitotenv.2016.11.025 27908624

[grl61575-bib-0007] Cui, Y. , Ji, D. , Maenhaut, W. , Gao, W. , Zhang, R. , & Wang, Y. (2020). Levels and sources of hourly PM2.5‐related elements during the control period of the COVID‐19 pandemic at a rural site between Beijing and Tianjin. Science of The Total Environment, 744, 140840 10.1016/j.scitotenv.2020.140840 PMC7347310 32674021

[grl61575-bib-0008] Dallmann, T. R. , DeMartini, S. J. , Kirchstetter, T. W. , Herndon, S. C. , Onasch, T. B. , Wood, E. C. , & Harley, R. A. (2012). On‐road measurement of gas and particle phase pollutant emission factors for individual heavy‐duty diesel trucks. Environmental Science & Technology, 46(15), 8511–8518. 10.1021/es301936c 22799607

[grl61575-bib-0009] Dallmann, T. R. , Kirchstetter, T. W. , DeMartini, S. J. , & Harley, R. A. (2013). Quantifying On‐Road Emissions from Gasoline‐Powered Motor Vehicles: Accounting for the Presence of Medium‐ and Heavy‐Duty Diesel Trucks. Environmental Science & Technology, 47(23), 13,873–13,881. 10.1021/es402875u 24215572

[grl61575-bib-0011] Ding, A. J. , Huang, X. , Nie, W. , Sun, J. N. , Kerminen, V. M. , Petäjä, T. , Su, H. , Cheng, Y. F. , Yang, X. Q. , Wang, M. H. , Chi, X. G. , Wang, J. P. , Virkkula, A. , Guo, W. D. , Yuan, J. , Wang, S. Y. , Zhang, R. J. , Wu, Y. F. , Song, Y. , Zhu, T. , Zilitinkevich, S. , Kulmala, M. , & Fu, C. B. (2016). Enhanced haze pollution by black carbon in megacities in China. Geophysical Research Letters, 43, 2873–2879. 10.1002/2016GL067745

[grl61575-bib-0012] Fang, W. , Du, K. , Andersson, A. , Xing, Z. , Cho, C. , Kim, S.‐W. , Deng, J. , & Gustafsson, Ö. (2018). Dual‐Isotope Constraints on Seasonally Resolved Source Fingerprinting of Black Carbon Aerosols in Sites of the Four Emission Hot Spot Regions of China. Journal of Geophysical Research: Atmospheres, 123(20), 11,735–711,747. 10.1029/2018JD028607

[grl61575-bib-0014] Grange, S. K. , & Carslaw, D. C. (2019). Using meteorological normalisation to detect interventions in air quality time series. Science of the Total Environment, 653, 578–588. 10.1016/j.scitotenv.2018.10.344 30759588

[grl61575-bib-0015] Grange, S. K. , Carslaw, D. C. , Lewis, A. C. , Boleti, E. , & Hueglin, C. (2018). Random forest meteorological normalisation models for Swiss PM10 trend analysis. Atmospheric Chemistry and Physics, 18(9), 6223–6239. 10.5194/acp-18-6223-2018

[grl61575-bib-0016] Hansen, A. D. A. (2005). The Aethalometer. Berkeley, CA: Magee Scientific Company.

[grl61575-bib-0017] Harrison, R. M. , Beddows, D. C. S. , Jones, A. M. , Calvo, A. , Alves, C. , & Pio, C. (2013). An evaluation of some issues regarding the use of aethalometers to measure woodsmoke concentrations. Atmospheric Environment, 80, 540–548. 10.1016/j.atmosenv.2013.08.026

[grl61575-bib-0018] Huang, X. , Ding, A. , Gao, J. , Zheng, B. , Zhou, D. , Qi, X. , Tang, R. , Wang, J. , Ren, C. , Nie, W. , Chi, X. , Xu, Z. , Chen, L. , Li, Y. , Che, F. , Pang, N. , Wang, H. , Tong, D. , Qin, W. , Cheng, W. , Liu, W. , Fu, Q. , Liu, B. , Chai, F. , Davis, S. J. , Zhang, Q. , & He, K. (2020). Enhanced secondary pollution offset reduction of primary emissions during COVID‐19 lockdown in China. National Science Review. 10.1093/nsr/nwaa137 PMC7337733 34676092

[grl61575-bib-0019] Janssen, N. A. H. , Hoek, G. , Simic‐Lawson, M. , Fischer, P. , van Bree, L. , ten Brink, H. , Keuken, M. , Atkinson, R. W. , Anderson, H. R. , Brunekreef, B. , & Cassee, F. R. (2011). Black carbon as an additional Indicator of the Adverse Health Effects of Airborne Particles Compared with PM10 and PM2.5. Environmental Health Perspectives, 119(12), 1691–1699. 10.1289/ehp.1003369 21810552 PMC3261976

[grl61575-bib-0020] Jiang, Y. , Liu, X. , Yang, X.‐Q. , & Wang, M. (2013). A numerical study of the effect of different aerosol types on East Asian summer clouds and precipitation. Atmospheric Environment, 70, 51–63. 10.1016/j.atmosenv.2012.12.039

[grl61575-bib-0023] Lelieveld, J. , Pozzer, A. , Pöschl, U. , Fnais, M. , Haines, A. , & Münzel, T. (2020). Loss of life expectancy from air pollution compared to other risk factors: A worldwide perspective. Cardiovascular Research, 116(11), 1910–1917. 10.1093/cvr/cvaa025 32123898 PMC7449554

[grl61575-bib-0024] Li, K. , Chen, L. , White, S. J. , Zheng, X. , Lv, B. , Lin, C. , Bao, Z. , Wu, X. , Gao, X. , Ying, F. , Shen, J. , Azzi, M. , & Cen, K. (2018). Chemical characteristics and sources of PM1 during the 2016 summer in Hangzhou. Environmental Pollution, 232, 42–54. 10.1016/j.envpol.2017.09.016 28935404

[grl61575-bib-0025] Liu, D. , Whitehead, J. , Alfarra, M. R. , Reyes‐Villegas, E. , Spracklen, D. V. , Reddington, C. L. , Kong, S. , Williams, P. I. , Ting, Y. C. , Haslett, S. , Taylor, J. W. , Flynn, M. J. , Morgan, W. T. , McFiggans, G. , Coe, H. , & Allan, J. D. (2017). Black‐carbon absorption enhancement in the atmosphere determined by particle mixing state. Nature Geoscience, 10(3), 184–188. 10.1038/ngeo2901

[grl61575-bib-0026] Liu, L. , Kong, S. , Zhang, Y. , Wang, Y. , Xu, L. , Yan, Q. , Lingaswamy, A. P. , Shi, Z. , Lv, S. , Niu, H. , Shao, L. , Hu, M. , Zhang, D. , Chen, J. , Zhang, X. , & Li, W. (2017). Morphology, composition, and mixing state of primary particles from combustion sources—Crop residue, wood, and solid waste. Scientific Reports, 7(1), 5047 10.1038/s41598-017-05357-2 28698671 PMC5505958

[grl61575-bib-0027] Liu, Y. , Yan, C. , & Zheng, M. (2018). Source apportionment of black carbon during winter in Beijing. Science of the Total Environment, 618, 531–541. 10.1016/j.scitotenv.2017.11.053 29149737

[grl61575-bib-0029] Okuda, T. , Matsuura, S. , Yamaguchi, D. , Umemura, T. , Hanada, E. , Orihara, H. , Tanaka, S. , He, K. , Ma, Y. , Cheng, Y. , & Liang, L. (2011). The impact of the pollution control measures for the 2008 Beijing Olympic Games on the chemical composition of aerosols. Atmospheric Environment, 45(16), 2789–2794. 10.1016/j.atmosenv.2011.01.053

[grl61575-bib-0030] Sandradewi, J. , Prévôt, A. S. H. , Szidat, S. , Perron, N. , Alfarra, M. R. , Lanz, V. A. , Weingartner, E. , & Baltensperger, U. (2008). Using aerosol light absorption measurements for the quantitative determination of wood burning and traffic emission contributions to particulate matter. Environmental Science & Technology, 42(9), 3316–3323. 10.1021/es702253m 18522112

[grl61575-bib-0031] Sandradewi, J. , Prévôt, A. S. H. , Weingartner, E. , Schmidhauser, R. , Gysel, M. , & Baltensperger, U. (2008). A study of wood burning and traffic aerosols in an Alpine valley using a multi‐wavelength Aethalometer. Atmospheric Environment, 42(1), 101–112. 10.1016/j.atmosenv.2007.09.034

[grl61575-bib-0032] State Council of the People's Republic of China . (2013). Notice of the General Office of The State Council on issuing the air pollution prevention and control action plan, http://www.gov.cn/zwgk/2013‐09/12/content_2486773.htm

[grl61575-bib-0033] Sun, Y. , Wang, Z. , Wild, O. , Xu, W. , Chen, C. , Fu, P. , du, W. , Zhou, L. , Zhang, Q. , Han, T. , Wang, Q. , Pan, X. , Zheng, H. , Li, J. , Guo, X. , Liu, J. , & Worsnop, D. R. (2016). “APEC Blue”: Secondary aerosol reductions from emission controls in Beijing. Scientific Reports, 6(1), 20668 10.1038/srep20668 26891104 PMC4758222

[grl61575-bib-0034] Tian, H. , Liu, Y. , Li, Y. , Wu, C.‐H. , Chen, B. , Kraemer, M. U. G. , Li, B. , Cai, J. , Xu, B. , Yang, Q. , Wang, B. , Yang, P. , Cui, Y. , Song, Y. , Zheng, P. , Wang, Q. , Bjornstad, O. N. , Yang, R. , Grenfell, B. T. , Pybus, O. G. , & Dye, C. (2020). An investigation of transmission control measures during the first 50 days of the COVID‐19 epidemic in China. Science, 368(6491), 638–642. 10.1126/science.abb6105 32234804 PMC7164389

[grl61575-bib-0036] Vu, T. V. , Shi, Z. , Cheng, J. , Zhang, Q. , He, K. , Wang, S. , & Harrison, R. M. (2019). Assessing the impact of clean air action on air quality trends in Beijing using a machine learning technique. Atmospheric Chemistry and Physics, 19(17), 11,303–11,314. 10.5194/acp-19-11303-2019

[grl61575-bib-0037] Wang, J. , Lei, Y. , & Ning, M. (2018). Chinese model for improving air quality: An assessment of action plan of air pollution prevention and control (in Chinese). Environmental Protection, 46(2), 7–11.

[grl61575-bib-0038] Wang, P. , Chen, K. , Zhu, S. , Wang, P. , & Zhang, H. (2020). Severe air pollution events not avoided by reduced anthropogenic activities during COVID‐19 outbreak. Resources, Conservation and Recycling, 158, 104814 10.1016/j.resconrec.2020.104814 PMC7151380 32300261

[grl61575-bib-0039] Wang, Y. , Liu, F. , He, C. , Bi, L. , Cheng, T. , Wang, Z. , Zhang, H. , Zhang, X. , Shi, Z. , & Li, W. (2017). Fractal dimensions and mixing structures of soot particles during atmospheric processing. Environmental Science & Technology Letters, 4(11), 487–493. 10.1021/acs.estlett.7b00418

[grl61575-bib-0040] Weingartner, E. , Saathoff, H. , Schnaiter, M. , Streit, N. , Bitnar, B. , & Baltensperger, U. (2003). Absorption of light by soot particles: Determination of the absorption coefficient by means of aethalometers. Journal of Aerosol Science, 34(10), 1445–1463. 10.1016/S0021-8502(03)00359-8

[grl61575-bib-0041] WHO . (2020). WHO timeline—COVID‐19, https://www.who.int/news-room/detail/27‐04‐2020‐who‐timeline‐‐‐covid‐19

[grl61575-bib-0042] Wu, C. , Wu, D. , & Yu, J. Z. (2018). Quantifying black carbon light absorption enhancement with a novel statistical approach. Atmospheric Chemistry and Physics, 18(1), 289–309. 10.5194/acp-18-289-2018

[grl61575-bib-0043] Wu, J. , Xu, C. , Wang, Q. , & Cheng, W. (2016). Potential sources and formations of the PM2.5 pollution in urban Hangzhou. Atmosphere, 7(8), 100 10.3390/atmos7080100

[grl61575-bib-0044] Yuan, Q. , Qi, B. , Hu, D. , Wang, J. , Zhang, J. , Yang, H. , Zhang, S. , Liu, L. , Xu, L. , & Li, W. (2021). Spatiotemporal variations and reduction of air pollutants during the COVID‐19 pandemic in a megacity of Yangtze River Delta in China. Science of the Total Environment, 751, 141820 10.1016/j.scitotenv.2020.141820 PMC7440035 32861951

[grl61575-bib-0045] Zhang, R. , Wang, G. , Guo, S. , Zamora, M. L. , Ying, Q. , Lin, Y. , Wang, W. , Hu, M. , & Wang, Y. (2015). Formation of urban fine particulate matter. Chemical Reviews, 115(10), 3803–3855. 10.1021/acs.chemrev.5b00067 25942499

[grl61575-bib-0046] Zhang, R. , Zhang, Y. , Lin, H. , Feng, X. , Fu, T.‐M. , & Wang, Y. (2020). NO*x* emission reduction and recovery during COVID‐19 in East China. Atmosphere, 11(4), 433 10.3390/atmos11040433

[grl61575-bib-0047] Zhang, X. Y. , Wang, J. Z. , Wang, Y. Q. , Liu, H. L. , Sun, J. Y. , & Zhang, Y. M. (2015). Changes in chemical components of aerosol particles in different haze regions in China from 2006 to 2013 and contribution of meteorological factors. Atmospheric Chemistry and Physics, 15(22), 12,935–12,952. 10.5194/acp-15-12935-2015

[grl61575-bib-0048] Zhang, Y. , Li, X. , Li, M. , Zheng, Y. , Geng, G. , Hong, C. , Li, H. , Tong, D. , Zhang, X. , Cheng, Y. , Su, H. , He, K. , & Zhang, Q. (2018). Reduction in black carbon light absorption due to multi‐pollutant emission control during APEC China 2014. Atmospheric Chemistry and Physics, 18(14), 10,275–10,287. 10.5194/acp-18-10275-2018

[grl61575-bib-0049] Zhang, Y. , Yuan, Q. , Huang, D. , Kong, S. , Zhang, J. , Wang, X. , Lu, C. , Shi, Z. , Zhang, X. , Sun, Y. , Wang, Z. , Shao, L. , Zhu, J. , & Li, W. (2018). Direct observations of fine primary particles from residential coal burning: Insights into their morphology, composition, and hygroscopicity. Journal of Geophysical Research: Atmospheres, 123(22), 12,964–12,979. 10.1029/2018JD028988

[grl61575-bib-0050] Zhao, J. , Du, W. , Zhang, Y. , Wang, Q. , Chen, C. , Xu, W. , Han, T. , Wang, Y. , Fu, P. , Wang, Z. , & Li, Z. (2017). Insights into aerosol chemistry during the 2015 China Victory Day parade: Results from simultaneous measurements at ground level and 260 m in Beijing. Atmospheric Chemistry and Physics, 17(4), 3215–3232. 10.5194/acp-17-3215-2017

[grl61575-bib-0051] Zheng, H. , Kong, S. , Wu, F. , Cheng, Y. , Niu, Z. , Zheng, S. , Yang, G. , Yao, L. , Yan, Q. , Wu, J. , Zheng, M. , Chen, N. , Xu, K. , Yan, Y. , Liu, D. , Zhao, D. , Zhao, T. , Bai, Y. , Li, S. , & Qi, S. (2019). Intra‐regional transport of black carbon between the south edge of the North China Plain and central China during winter haze episodes. Atmospheric Chemistry and Physics, 19(7), 4499–4516. 10.5194/acp-19-4499-2019

[grl61575-bib-0052] Zotter, P. , Herich, H. , Gysel, M. , El‐Haddad, I. , Zhang, Y. , Močnik, G. , Hüglin, C. , Baltensperger, U. , Szidat, S. , & Prévôt, A. S. (2017). Evaluation of the absorption Ångström exponents for traffic and wood burning in the Aethalometer‐based source apportionment using radiocarbon measurements of ambient aerosol. Atmospheric Chemistry and Physics, 17(6), 4229–4249. 10.5194/acp-17-4229-2017

[grl61575-bib-0004] Cappa, C. D. , Onasch, T. B. , Massoli, P. , Worsnop, D. R. , Bates, T. S. , Cross, E. S. , Davidovits, P. , Hakala, J. , Hayden, K. L. , Jobson, B. T. , Kolesar, K. R. , Lack, D. A. , Lerner, B. M. , Li, S. M. , Mellon, D. , Nuaaman, I. , Olfert, J. S. , Petaja, T. , Quinn, P. K. , Song, C. , Subramanian, R. , Williams, E. J. , & Zaveri, R. A. (2012). Radiative absorption enhancements due to the mixing state of atmospheric black carbon. Science, 337(6098), 1078–1081. 10.1126/science.1223447 22936774

[grl61575-bib-0010] Day, D. E. , Hand, J. L. , Carrico, C. M. , Engling, G. , & Malm, W. C. (2006). Humidification factors from laboratory studies of fresh smoke from biomass fuels. Journal of Geophysical Research, 111, D22202 10.1029/2006JD007221

[grl61575-bib-0013] Favez, O. , El Haddad, I. , Piot, C. , Boréave, A. , Abidi, E. , Marchand, N. , Jaffrezo, J. L. , Besombes, J. L. , Personnaz, M. B. , Sciare, J. , & Wortham, H. (2010). Inter‐comparison of source apportionment models for the estimation of wood burning aerosols during wintertime in an Alpine city (Grenoble, France). Atmospheric Chemistry and Physics, 10(12), 5295–5314. 10.5194/acp-10-5295-2010

[grl61575-bib-0021] Kirchstetter, T. W. , Novakov, T. , & Hobbs, P. V. (2004). Evidence that the spectral dependence of light absorption by aerosols is affected by organic carbon. Journal of Geophysical Research, 109, D21208 10.1029/2004JD004999

[grl61575-bib-0022] Lack, D. A. , & Langridge, J. M. (2013). On the attribution of black and brown carbon light absorption using the Ångström exponent. Atmospheric Chemistry and Physics, 13(20), 10,535–10,543. 10.5194/acp-13-10535-2013

[grl61575-bib-0028] Mousavi, A. , Sowlat, M. H. , Hasheminassab, S. , Polidori, A. , & Sioutas, C. (2018). Spatio‐temporal trends and source apportionment of fossil fuel and biomass burning black carbon (BC) in the Los Angeles Basin. Science of the Total Environment, 640–641, 1231–1240. 10.1016/j.scitotenv.2018.06.022 30021288

[grl61575-bib-0035] Tian, J. , Wang, Q. , Ni, H. , Wang, M. , Zhou, Y. , Han, Y. , Shen, Z. , Pongpiachan, S. , Zhang, N. , Zhao, Z. , Zhang, Q. , Zhang, Y. , Long, X. , & Cao, J. (2019). Emission characteristics of primary brown carbon absorption from biomass and coal burning: Development of an optical emission inventory for China. Journal of Geophysical Research: Atmospheres, 124(3), 1879–1893. 10.1029/2018JD029352

