# Supplementary material for: Variation in Concentration and Sources of Black Carbon in a Megacity of China During the COVID‐19 Pandemic
Source: Geophys Res Lett. 2020 Nov 28;47(23):e2020GL090444. doi: 10.1029/2020GL090444 (PMC7744912; doi:10.1029/2020GL090444)
Supplement: Supplementary file 1 — Supporting Information S1 [file GRL-47-e2020GL090444-s001.docx]

Supplementary Information for

**Variation in concentration and sources of black carbon in a megacity of China during the COVID-19 pandemic**

Liang Xu^1^, Jian Zhang^1^, Xin Sun^2^, Shengchen Xu^2^, Meng Shan^3^, Qi Yuan^1^, Lei Liu^1^, Zhenhong Du^1^, Dantong Liu^1^, Da Xu^2^, Congbo Song^4^, Bowen Liu^5^, Gongda Lu^4^, Zongbo Shi^4^, Weijun Li^1,*^

^1^Key Laboratory of Geoscience Big Data and Deep Resource of Zhejiang Province & Department of Atmospheric Sciences, School of Earth Sciences, Zhejiang University, Hangzhou 310027, China

^2^Zhejiang Ecological and Environmental Monitoring Center, Hangzhou, 310012, China

^3^Zhejiang Linan Atmospheric Background National Observation and Research Station, Hangzhou 311307, China

^4^School of Geography, Earth and Environmental Sciences, University of Birmingham, Birmingham, B15 2TT, UK

^5^Department of Economics, University of Birmingham, Birmingham, B15 2TT, UK

*Corresponding author: W. Li ([liweijun@zju.edu.cn](mailto:liweijun@zju.edu.cn))

**The machine-learning based random forest algorithm and the model performance**

Weather conditions change rapidly, causing variations in the concentration of air pollutants even when the emissions do not change. Here, we applied a machine-learning based random forest (RF) algorithm to decouple the effects of meteorological conditions. To do this, we firstly build random forest model for black carbon (BC) and for each year (Jan to March). Briefly, the random forest model was built independently to each period (Jan-March 2019, Jan-March 2020), each site in Hangzhou city. 70% of the original data were randomly selected to build the model, which was then evaluated with the rest (30%) of the dataset. Similar to [Grange et al. (2018)](#_ENREF_4) and [Vu et al. (2019)](#_ENREF_12), the weather normalization was conducted using the "rmweather" R package, available at: https://cran.r-project.org/web/packages/rmweather/index.html. We only normalized the weather data but not time variables, which is similar to the [Vu et al. (2019)](#_ENREF_12), and resampling from the whole study period, which is similar to [Grange et al. (2018)](#_ENREF_4). The improved method is more suitable for tracking emission changes. The input features for the model included time variables (i.e., Unix time, Julian day, day of the week, and hour of the day), meteorological data from surface observations (i.e., temperature, relative humidity, wind speed, wind direction, atmospheric pressure). The day of week was categorical variables while all others were numeric. Following [Vu et al. (2019)](#_ENREF_12), the parameters for the RF models are: a forest of 300 trees: n_tree=300; the number of variables that may split at each node: mtry=3; the minimum size of terminal nodes: min_node_size=3. For every weather normalization, the explanatory variables were resampled from the variables (excluding the time variables) without replacement and randomly allocated to a dependent variable observation. The 1000 predictions were then aggregated using the arithmetic mean and this was the deweathered concentrations.

As mentioned before, 70% of the original data were used to train the model, and the rest (30%) of the dataset were applied to test the model. Table S5 presented information on the performance of our model to reproduce observations based on a number of statistical measures. The model shows a good performance with the correlation coefficients (r^2^ ,0.78-0.83) between model-predicted and observed data for the testing data sets (30% of observation data).

**AAE selection and uncertainty estimation for the BC_bb_ and BC_ff_ concentrations**

The AAE of biomass burning is affected by the type of biomass, the combustion condition, and the internal mixing with non-absorbing materials, leading to a large range of AAE_bb_ (0.9-2.1) in the previous literature ([Day et al., 2006](#_ENREF_2)). In the present study, AAE_bb_ is set to 2.0, corresponding to a typical frequently-used value previously reported for biomass burning ([Day et al., 2006](#_ENREF_2); [Favez et al., 2010](#_ENREF_3); [Kirchstetter et al., 2004](#_ENREF_6)).

Here, we refered to previous studies to estimate the total uncertainty for the BC_bb_ and BC_ff_ contributions ([Lack and Langridge, 2013](#_ENREF_7); [Mousavi et al., 2018](#_ENREF_9); [Tian et al., 2019](#_ENREF_11)). It can be calculated based on the uncertainties of Aethalometer measurements and the Aethalometer model calculations.

First, we should consider experimental uncertainties. [Hansen (2005)](#_ENREF_5) reported the uncertainty of the Aethalometer AE31 was about ± 5%. Thus, Unc_abs_ is designated as ± 5% to represent uncertainty in the absorption measurements of the AE31. Converting the absorption values to BC mass concentrations will lead to additional uncertainty, which mainly driven by MAC value calculations from aerosol mixing state, which leads to the optical “lensing” effect (particle aging effect). [Cappa et al. (2012)](#_ENREF_1) investigated the enhancement in the optical absorptions of aged BC particles and reported an average BC absorption enhancement factor of 1.06±0.02. Thus, Unc_lens_ is designated as ± 6% to represent uncertainty from the lensing absorption enhancement.

Second, we need to consider uncertainties associated with the Aethalometer model and the BC fractions interference from brown carbon. Referring [Zotter et al. (2017)](#_ENREF_15), choosing AEE values of 1.0 and 2.0 for AAE_ff_ and AAE_bb_ will lead to about ~20% uncertainty in BC_bb_ and BC_ff_ concentrations. Thus, Unc_AAE_ is treated as 20%. Absorbing organic aerosol, especially absorbing secondary organic aerosol (SOA), also known as brown carbon (BrC), may affect the BC fractions assigned to biomass burning ([Lack and Langridge, 2013](#_ENREF_7)). [Lack and Langridge (2013)](#_ENREF_7) also reported an average disagreement of 34% to the BC absorption due to BrC. Thus, we applied 34% as the value of Unc_BrC_.

At the end, the total uncertainty for the model-derived BC_bb_ and BC_ff_ can be estimated as follows:

Unc_total_=$\left[ \left( \text{Unc}\text{abs} \right)\text{2}\text{+}\left( \text{Unc}\text{lens} \right)\text{2}\text{+}\left( \text{Unc}\text{AAE} \right)\text{2}\text{+}\left( \text{Unc}\text{BrC} \right)\text{2} \right]^{\frac{\text{1}}{\text{2}}}$

=$\left[ \left( \text{5\%} \right)\text{2}\text{+}\left( \text{6\%} \right)\text{2}\text{+}\left( \text{20\%} \right)\text{2}\text{+}\left( \text{34\%} \right)\text{2} \right]^{\frac{\text{1}}{\text{2}}}$

=40%

Thus, based on the above calculations, the model-derived BC fractions (BC_bb_ and BC_ff_) are associated with 40% uncertainty.

**Figures**


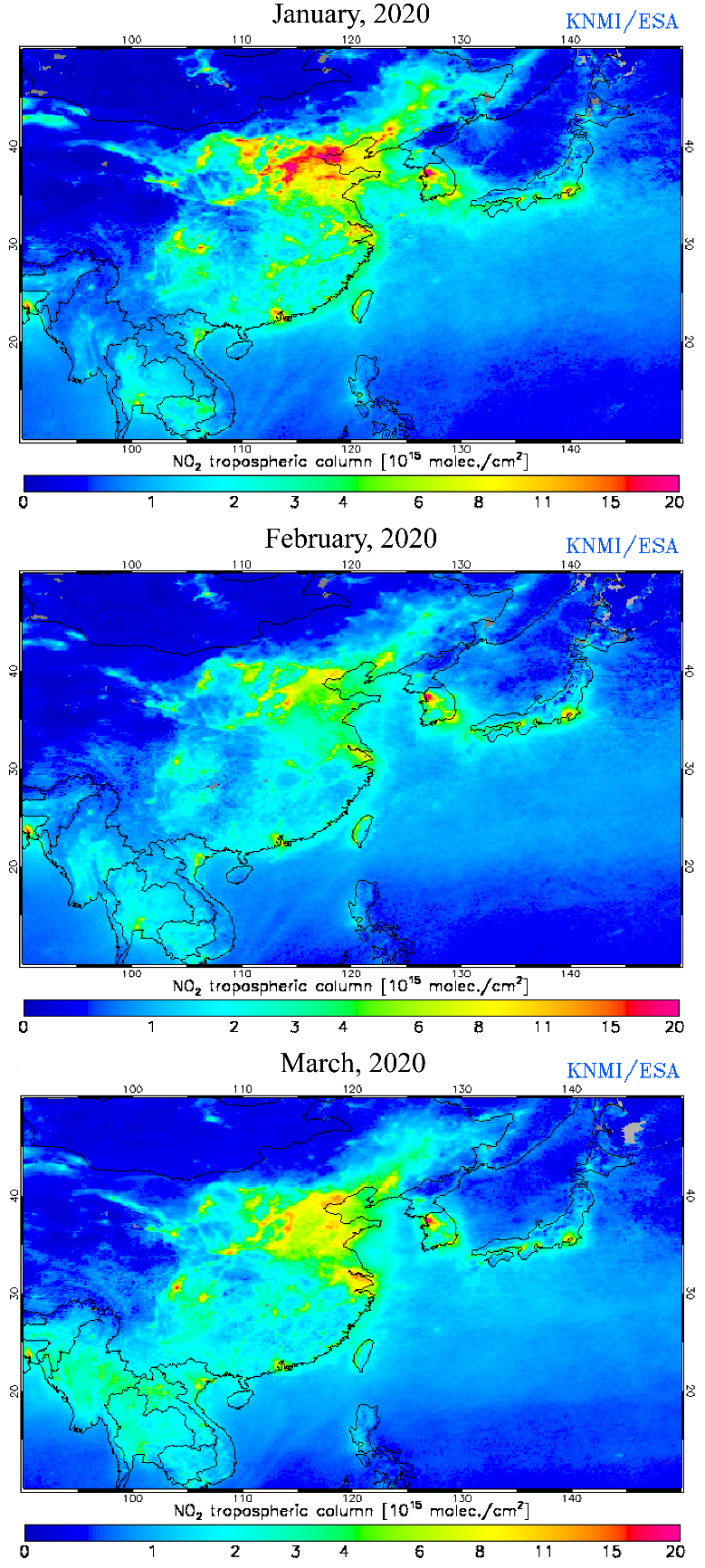


Figure S1. Monthly mean NO_2_ concentration of East Asia in January, February, and March, 2020 observed from TROPOMI sensor on board the Copernicus Sentinel-5 Precursor satellite. Data source: http://www.temis.nl/


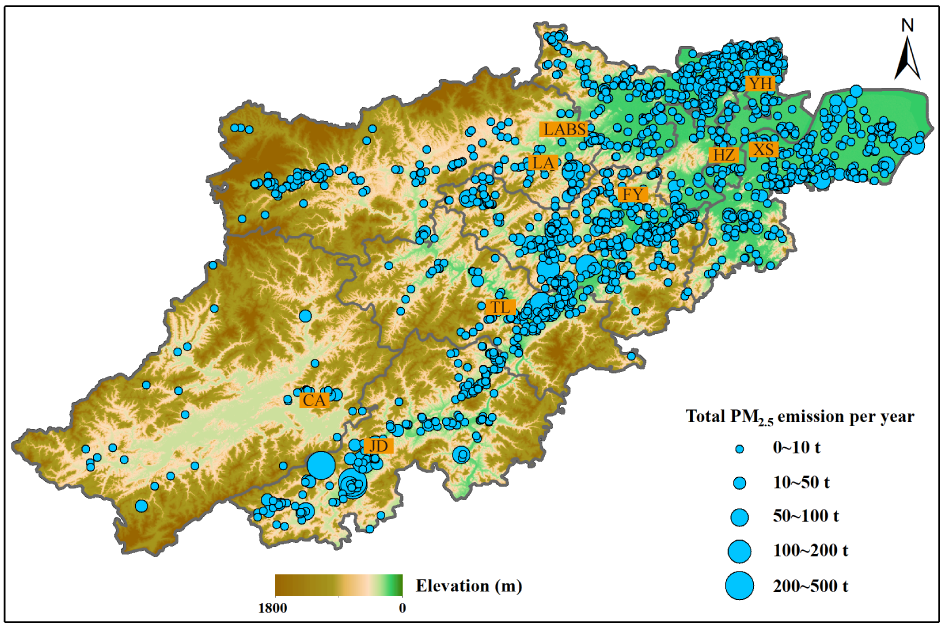


Figure S2. Distribution of enterprises with annual PM_2.5_ emission in Hangzhou


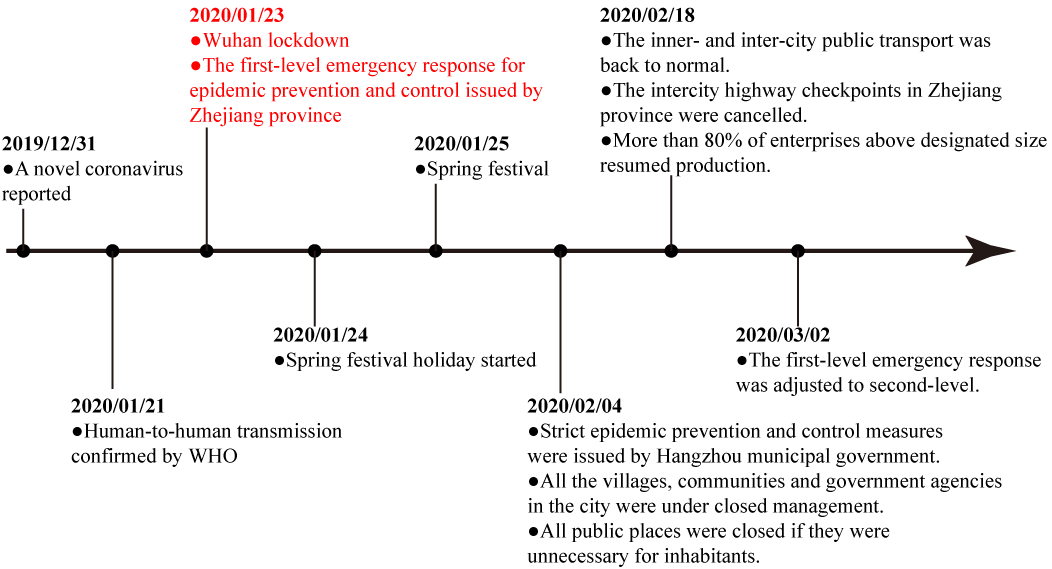


Figure S3. The timeline of COVID-19 outbreak and regulations issued by the government for epidemic control.


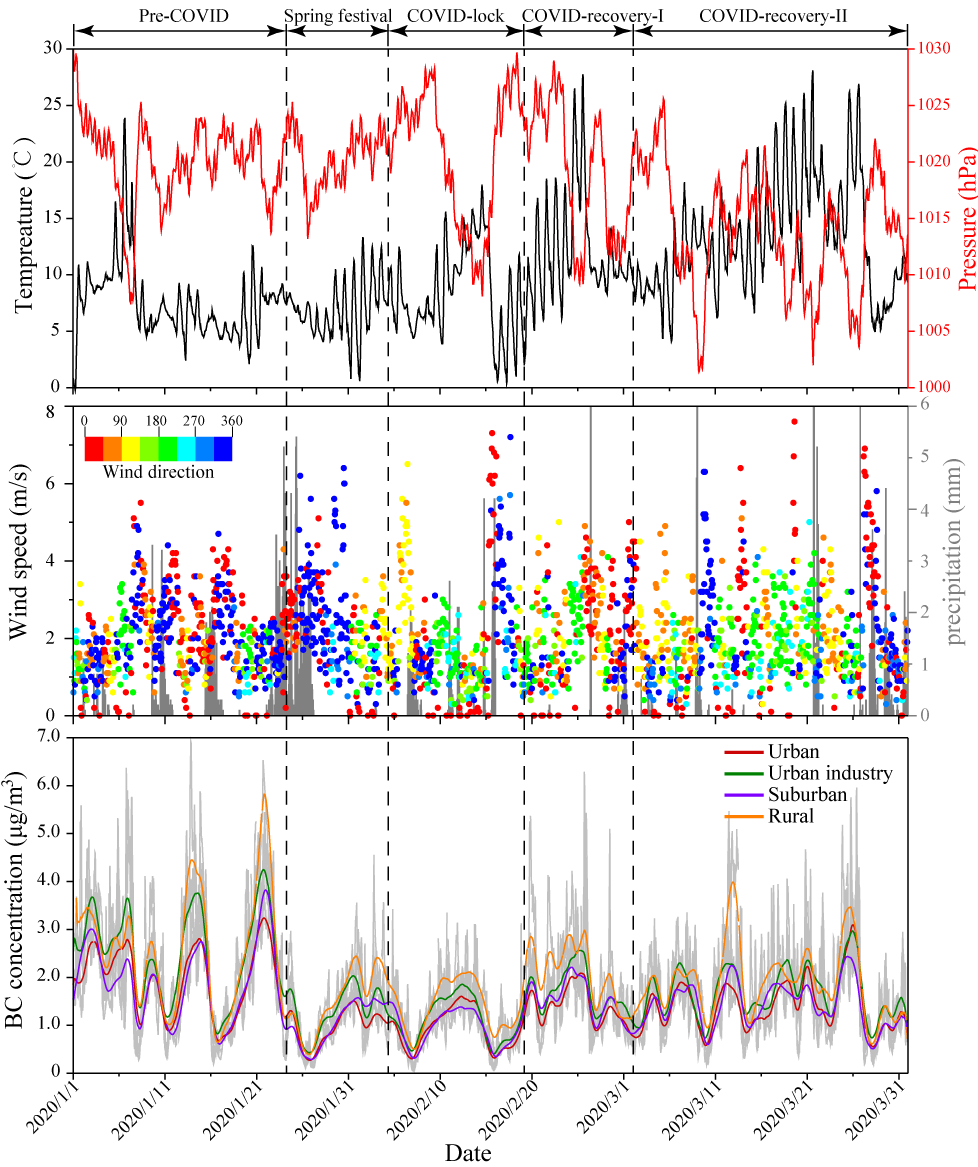


Figure S4. The time series of meteorological parameters, hourly BC concentrations (grey line) and 24-hour moving average (solid line) from 1 January to 31 March in Hangzhou.


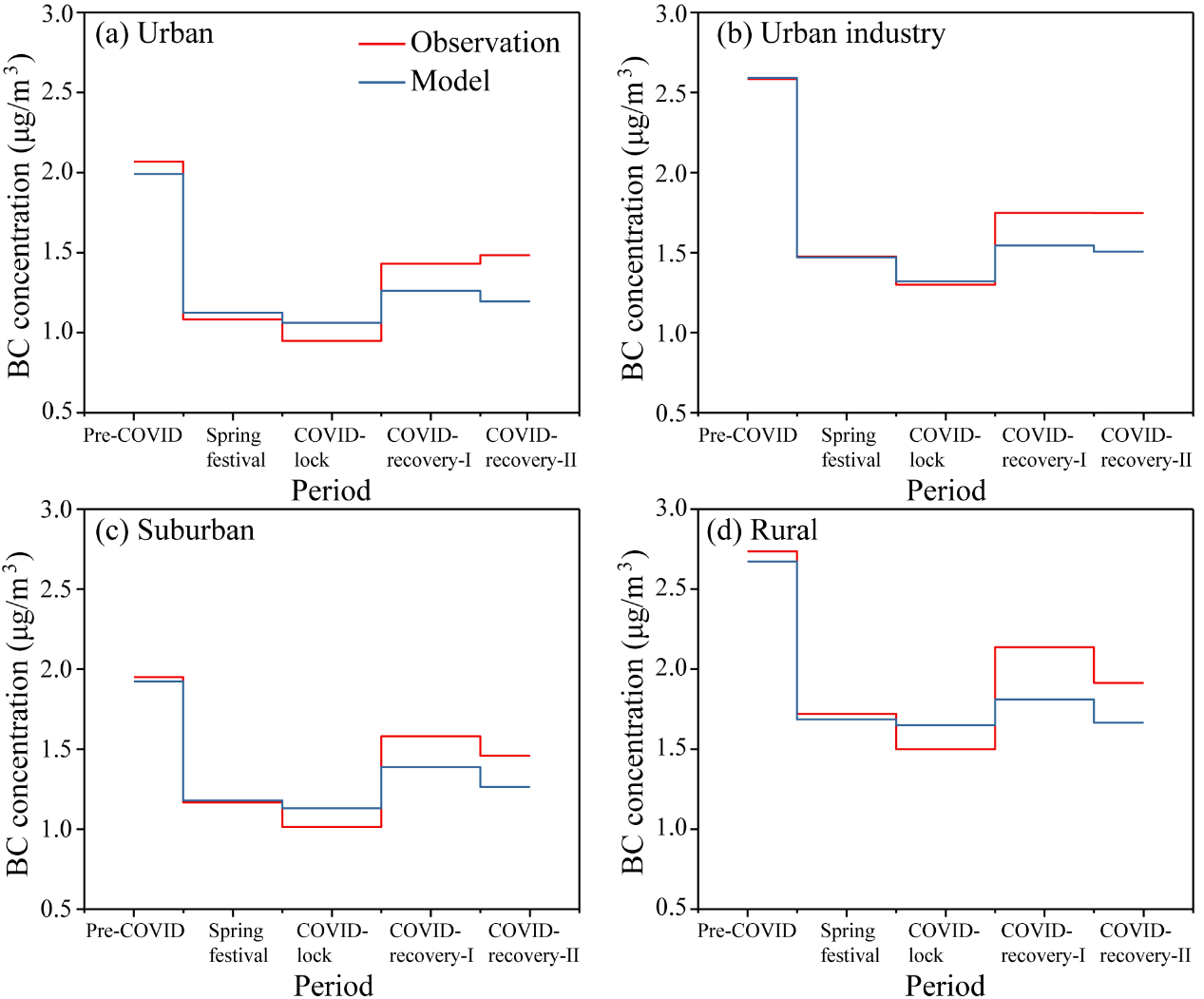


Figure S5. The observed and model deweathered BC concentration in different areas during the five stages.


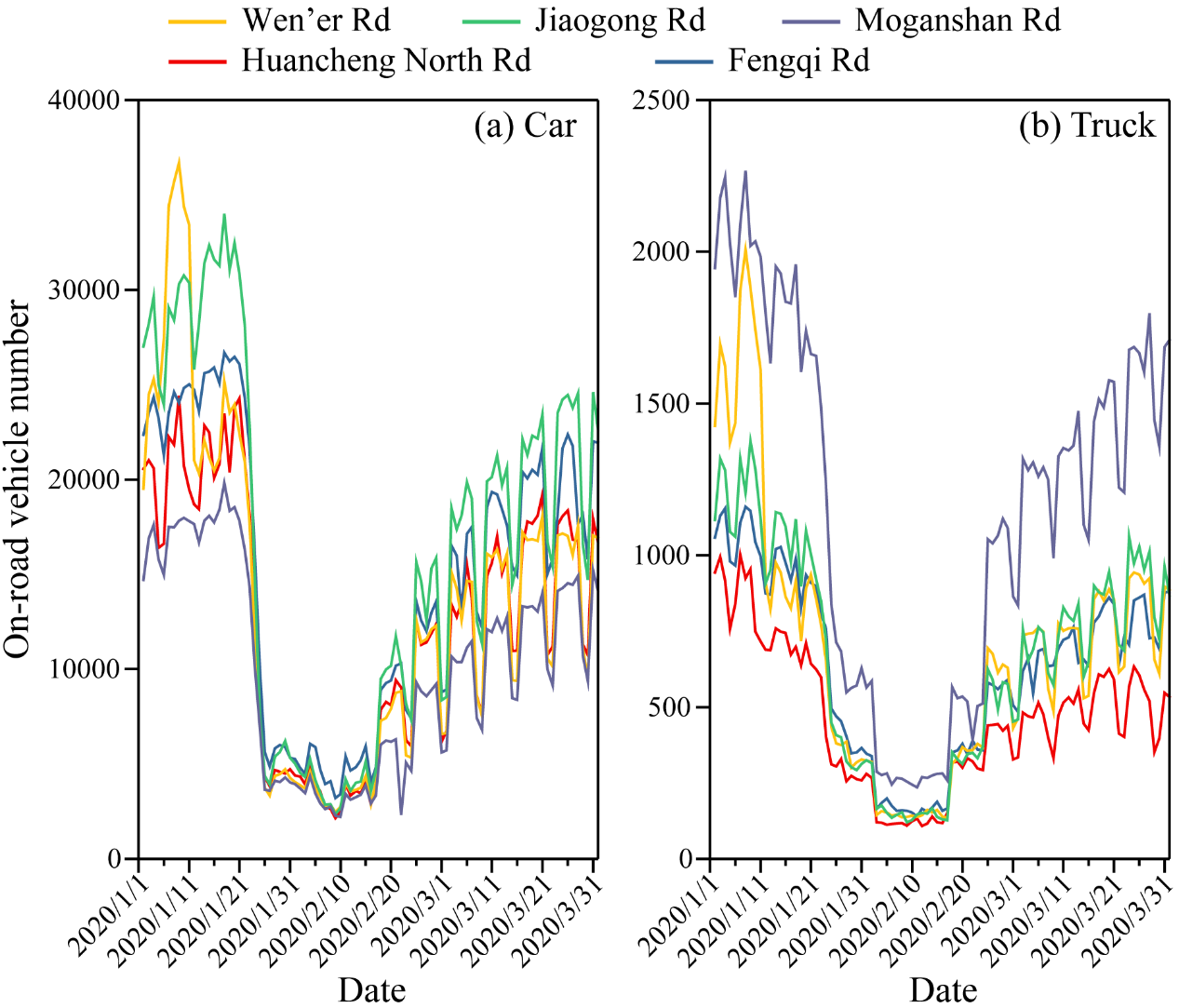


Figure S6. Number of different vehicles (car: gasoline engine, truck: diesel engine) running on five randomly selected roads in the urban area. Data source: Hangzhou Traffic Police Division.


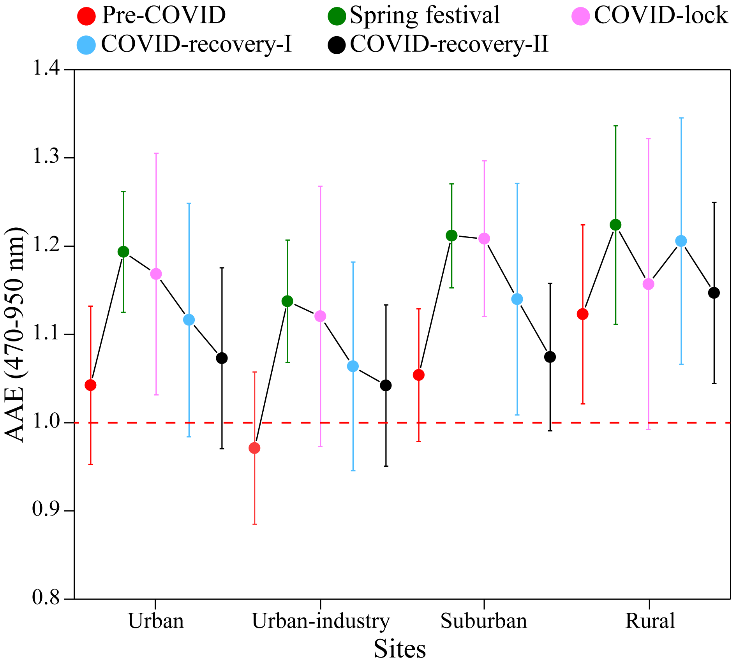


Figure S7. The absorption Ångström exponents (AAE) between 470 and 950 nm at four areas. Lines and error bars to show the mean ± σ AAE at each stage. The red dash line indicates the AAE of pure BC (AAE~1).


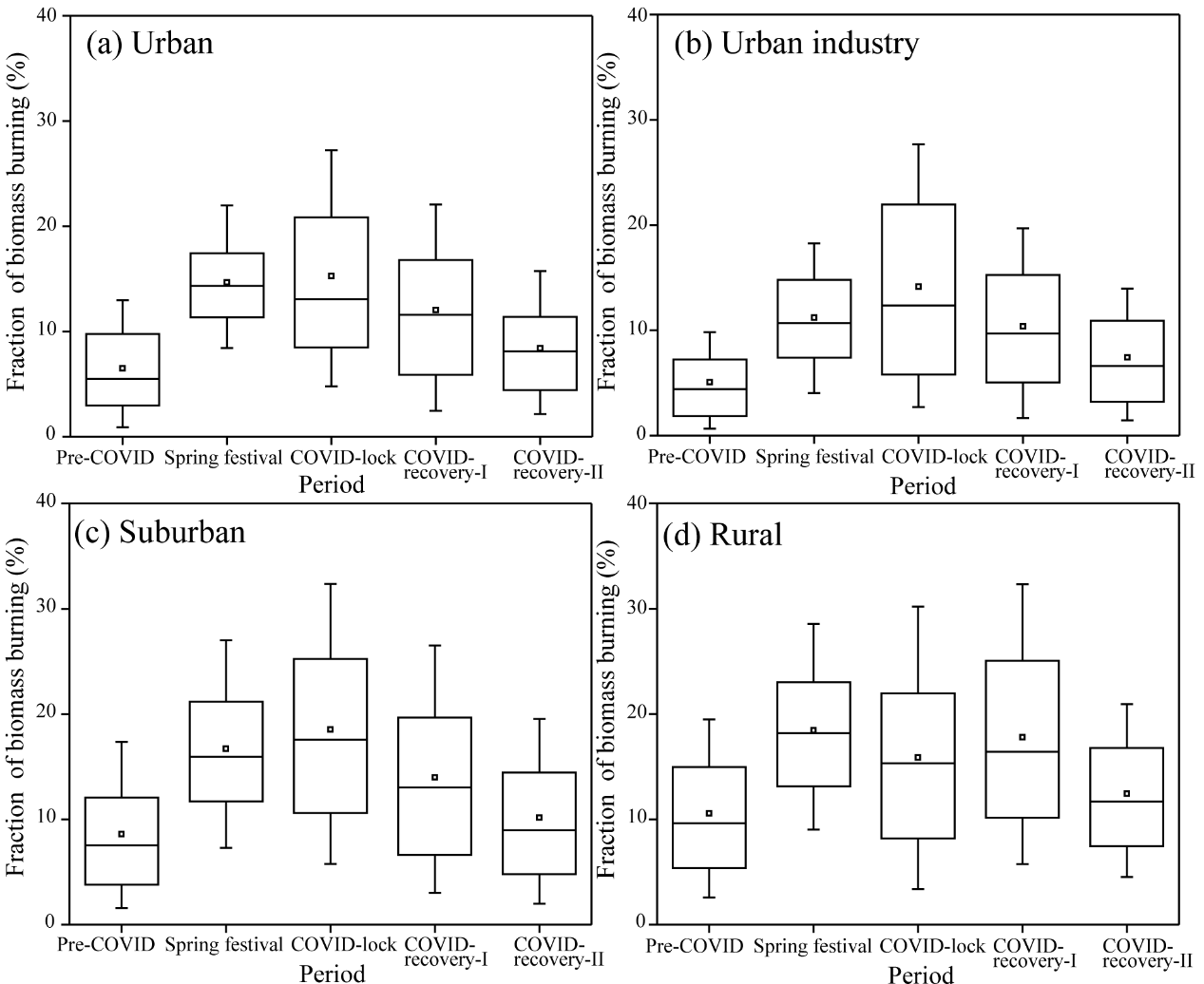


Figure S8. The relative contribution of biomass burning to total BC (f_bb_) on weekdays.


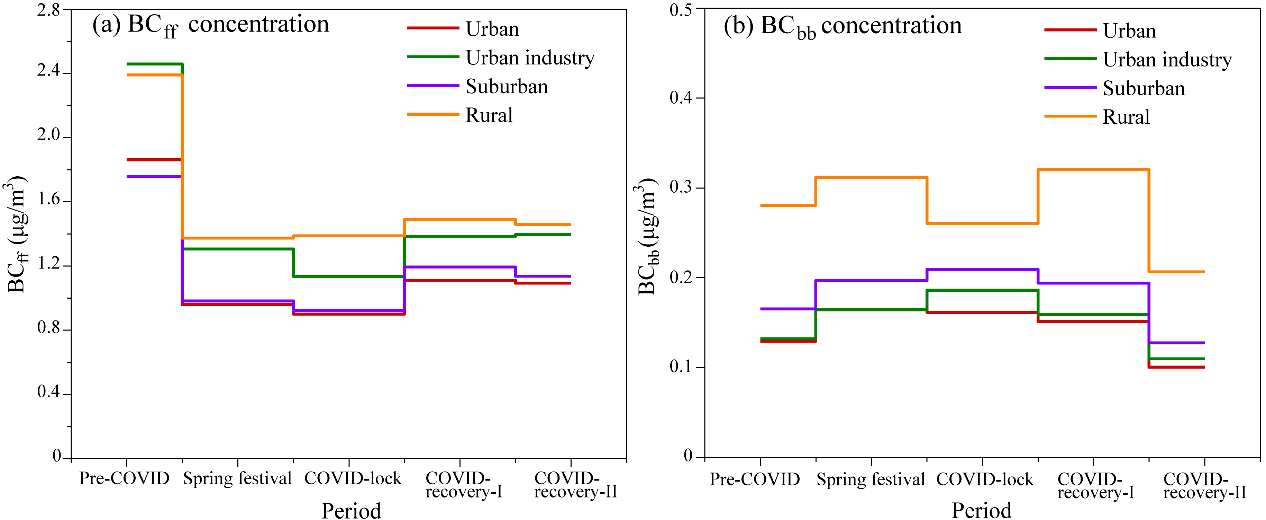


Figure S9 The deweathered BC concentration from biomass burning (BC_bb_) and fossil fuel (BC_ff_) on weekdays. The detailed data is presented in Table S4

**Tables**

Table S1. Information of observation sites. The data were obtained from the Statistic Yearbook of Hangzhou.

| **Category** | **Site** | **Location** | **Industry Number^1^** | **Gross industrial output value^1^** | **private vehicles number 2018** | **Days with good air quality^2^ 2018** |
| --- | --- | --- | --- | --- | --- | --- |
|  |  |  |  | billion Yuan | ×10^4^ unit |  |
| Urban | Hangzhou (HZ) | 30º14'N, 120º10'E | 490 | 348 | 101.8 | 269 |
| Urban industry | Xiaoshan (XS) | 30º11'N, 120º17'E | 1575 | 397 | 39.0 | 269 |
| Urban industry | Yuhang (YH) | 30º25'N, 120º17'E | 1155 | 180 | 33.2 | 269 |
| Suburban | Fuyang (FY) | 30º03'N, 119º57'E | 622 | 137 | 12.7 | 328 |
| Suburban | Lin'an (LA) | 30º13'N, 119º42'E | 598 | 81 | 11.9 | 324 |
| Suburban | Tonglu (TL) | 29º49'N, 119º41'E | 292 | 38 | 7.2 | 328 |
| Suburban | Chun'an (CA) | 29º37'N, 119º01'E | 85 | 10 | 5.4 | 331 |
| Suburban | Jiande (JD) | 29º29'N, 119º16'E | 308 | 41 | 6.7 | 346 |
| Rural | Lin'an Background Station (LABS) | 30º10'N, 119º26'E | - | - | - | 324 |

^1^ Industrial enterprises above designated size (2018)

^2^ Air quality index (AQI) ≤100

Table S2. Comparisons in BC concentrations between Hangzhou COVID lockdown and other major events over China.

| **Site** | **Event** | **Period** | **Mean value (μg/m3)** | **Change perc (%)** | **References** |
| --- | --- | --- | --- | --- | --- |
| Hangzhou | COVID-19 lockdown 2020/01/01-03/31 | pre-COVID | 2.30 | - | This study |
|  |  | COVID-lock | 1.30 | -44% |  |
|  |  | COVID-recovery | 1.45 | +12% |  |
| Urban Beijing | The 2008 Beijing Olympic Games 2008/07/01-09/20 | During | 3.2 | - | [Okuda et al. (2011)](#_ENREF_10) |
| Urban Beijing | APEC 2014/10/28-11/21 | Before APEC | 3.4 | - | [Zhang et al. (2018)](#_ENREF_13) |
|  |  | During APEC | 2.5 | -27% |  |
|  |  | After APEC | 6.0 | +58% |  |
| Urban Beijing | The 2015 China Victory Day parade 2015/08/22-09/30 | During | 1.2 | - | [Zhao et al. (2017)](#_ENREF_14) |
|  |  | After | 2.7 | +57% |  |
| Urban Hangzhou | The 2016 G20 summit 2016/08/05-09/23 | Before G20 | 2.2 | - | [Li et al. (2018)](#_ENREF_8) |
|  |  | During G20 | 1.7 | -23% |  |
|  |  | After G20 | 2.9 | +41% |  |

Table S3. Average deweathered BC concentration (μg/m^3^) in different sites in different period

| **Category** | **Pre-COVID** | | **Spring festival** | | **COVID-lock** | | **COVID-recovery-I** | | **COVID-recovery-II** | |
| --- | --- | --- | --- | --- | --- | --- | --- | --- | --- | --- |
|  | **BC (μg/m^3^)** | **Change^1^ (%)** | **BC (μg/m^3^)** | **Change (%)** | **BC (μg/m^3^)** | **Change (%)** | **BC (μg/m^3^)** | **Change (%)** | **BC (μg/m^3^)** | **Change (%)** |
| Urban | 1.99 | - | 1.13 | -43% | 1.06 | -47% | 1.26 | -37% | 1.19 | -40% |
| Urban industry | 2.59 | - | 1.47 | -43% | 1.32 | -49% | 1.54 | -40% | 1.51 | -42% |
| Suburban | 1.92 | - | 1.18 | -39% | 1.13 | -41% | 1.39 | -28% | 1.26 | -34% |
| Rural | 2.67 | - | 1.69 | -37% | 1.65 | -38% | 1.81 | -32% | 1.67 | -38% |

^1^ The relative change between different stages and the pre-COVID.

Table S4. Average deweathered BC concentration (μg/m^3^) from fossil fuel (BC_ff_) and biomass burning (BC_bb_).

| **Category** | **Pre-COVID** | | **Spring festival** | | **COVID-lock** | | **COVID-recovery-I** | | **COVID-recovery-II** | |
| --- | --- | --- | --- | --- | --- | --- | --- | --- | --- | --- |
|  | BC_ff_ | BC_bb_ | BC_ff_ | BC_bb_ | BC_ff_ | BC_bb_ | BC_ff_ | BC_bb_ | BC_ff_ | BC_bb_ |
| Urban | 1.86 | 0.13 | 0.96 | 0.16 | 0.90 | 0.16 | 1.11 | 0.15 | 1.09 | 0.10 |
| Urban industry | 2.46 | 0.13 | 1.31 | 0.16 | 1.13 | 0.19 | 1.39 | 0.16 | 1.40 | 0.11 |
| Suburban | 1.76 | 0.17 | 0.98 | 0.20 | 0.92 | 0.21 | 1.19 | 0.19 | 1.14 | 0.13 |
| Rural | 2.39 | 0.28 | 1.37 | 0.31 | 1.39 | 0.26 | 1.49 | 0.32 | 1.46 | 0.21 |

Table S5. The deweather model performance for testing data set (in hourly time resolution)

|  | n | FAC2 | MB | MGE | NMB | NMGE | RMSE | r^2^ | COE | IOA |
| --- | --- | --- | --- | --- | --- | --- | --- | --- | --- | --- |
| Chun_an | 653 | 0.93 | -3.77 | 249.60 | 0.00 | 0.21 | 351.31 | 0.78 | 0.36 | 0.68 |
| Hangzhou | 650 | 0.97 | -6.40 | 267.91 | 0.00 | 0.18 | 363.19 | 0.82 | 0.51 | 0.75 |
| Xiaoshan | 655 | 0.96 | -19.94 | 314.84 | -0.01 | 0.20 | 438.06 | 0.79 | 0.46 | 0.73 |
| Yuhang | 653 | 0.94 | 9.42 | 406.09 | 0.00 | 0.20 | 561.16 | 0.79 | 0.44 | 0.72 |
| Fuyang | 650 | 0.96 | 31.39 | 339.50 | 0.02 | 0.19 | 481.30 | 0.83 | 0.51 | 0.75 |
| Linan | 654 | 0.94 | 4.24 | 317.63 | 0.00 | 0.23 | 438.95 | 0.80 | 0.38 | 0.69 |
| Tonglu | 653 | 0.94 | -15.71 | 365.86 | -0.01 | 0.20 | 479.93 | 0.80 | 0.37 | 0.69 |
| Jiande | 651 | 0.96 | -1.56 | 193.38 | 0.00 | 0.18 | 260.47 | 0.83 | 0.49 | 0.75 |
| LABS | 639 | 0.96 | 31.06 | 399.12 | 0.02 | 0.20 | 608.58 | 0.82 | 0.43 | 0.72 |

Note: n (number of testing data), FAC2 (fraction of predictions with a factor of two), MB (mean bias), MGE (mean gross error), NMB (normalised mean bias), NMGE (normalised mean gross error), RMSE (root-mean-square error), r^2^ (correlation coefficients), COE (Coefficient of Efficiency), IOA (Index of Agreement)

**References**

Cappa, C. D., Onasch, T. B., Massoli, P., Worsnop, D. R., Bates, T. S., Cross, E. S., et al. (2012). Radiative Absorption Enhancements Due to the Mixing State of Atmospheric Black Carbon. *Science*, *337*(6098), 1078-1081. https://doi.org/10.1126/science.1223447

Day, D. E., Hand, J. L., Carrico, C. M., Engling, G., & Malm, W. C. (2006). Humidification factors from laboratory studies of fresh smoke from biomass fuels. *Journal of Geophysical Research: Atmospheres*, *111*(D22). https://doi.org/10.1029/2006JD007221

Favez, O., El Haddad, I., Piot, C., Boréave, A., Abidi, E., Marchand, N., et al. (2010). Inter-comparison of source apportionment models for the estimation of wood burning aerosols during wintertime in an Alpine city (Grenoble, France). *Atmospheric Chemistry and Physics*, *10*(12), 5295-5314. https://doi.org/10.5194/acp-10-5295-2010

Grange, S. K., Carslaw, D. C., Lewis, A. C., Boleti, E., & Hueglin, C. (2018). Random forest meteorological normalisation models for Swiss PM10 trend analysis. *Atmospheric Chemistry and Physics*, *18*(9), 6223-6239. https://doi.org/10.5194/acp-18-6223-2018

Hansen, A. D. A. (2005). The Aethalometer, Magee Scientific Company.

Kirchstetter, T. W., Novakov, T., & Hobbs, P. V. (2004). Evidence that the spectral dependence of light absorption by aerosols is affected by organic carbon. *Journal of Geophysical Research: Atmospheres*, *109*(D21). https://doi.org/10.1029/2004JD004999

Lack, D. A., & Langridge, J. M. (2013). On the attribution of black and brown carbon light absorption using the Ångström exponent. *Atmospheric Chemistry and Physics*, *13*(20), 10535-10543. https://doi.org/10.5194/acp-13-10535-2013

Li, K., Chen, L., White, S. J., Zheng, X., Lv, B., Lin, C., et al. (2018). Chemical characteristics and sources of PM1 during the 2016 summer in Hangzhou. *Environmental Pollution*, *232*, 42-54. https://doi.org/10.1016/j.envpol.2017.09.016

Mousavi, A., Sowlat, M. H., Hasheminassab, S., Polidori, A., & Sioutas, C. (2018). Spatio-temporal trends and source apportionment of fossil fuel and biomass burning black carbon (BC) in the Los Angeles Basin. *Science of The Total Environment*, *640-641*, 1231-1240. https://doi.org/https://doi.org/10.1016/j.scitotenv.2018.06.022

Okuda, T., Matsuura, S., Yamaguchi, D., Umemura, T., Hanada, E., Orihara, H., et al. (2011). The impact of the pollution control measures for the 2008 Beijing Olympic Games on the chemical composition of aerosols. *Atmospheric Environment*, *45*(16), 2789-2794. https://doi.org/10.1016/j.atmosenv.2011.01.053

Tian, J., Wang, Q., Ni, H., Wang, M., Zhou, Y., Han, Y., et al. (2019). Emission Characteristics of Primary Brown Carbon Absorption From Biomass and Coal Burning: Development of an Optical Emission Inventory for China. *Journal of Geophysical Research: Atmospheres*, *124*(3), 1879-1893. https://doi.org/10.1029/2018JD029352

Vu, T. V., Shi, Z., Cheng, J., Zhang, Q., He, K., Wang, S., & Harrison, R. M. (2019). Assessing the impact of clean air action on air quality trends in Beijing using a machine learning technique. *Atmospheric Chemistry and Physics*, *19*(17), 11303-11314. https://doi.org/10.5194/acp-19-11303-2019

Zhang, Y., Li, X., Li, M., Zheng, Y., Geng, G., Hong, C., et al. (2018). Reduction in black carbon light absorption due to multi-pollutant emission control during APEC China 2014. *Atmospheric Chemistry and Physics*, *18*(14), 10275-10287. https://doi.org/10.5194/acp-18-10275-2018

Zhao, J., Du, W., Zhang, Y., Wang, Q., Chen, C., Xu, W., et al. (2017). Insights into aerosol chemistry during the 2015 China Victory Day parade: results from simultaneous measurements at ground level and 260 m in Beijing. *Atmospheric Chemistry and Physics*, *17*(4), 3215-3232. https://doi.org/10.5194/acp-17-3215-2017

Zotter, P., Herich, H., Gysel, M., El-Haddad, I., Zhang, Y., Močnik, G., et al. (2017). Evaluation of the absorption Ångström exponents for traffic and wood burning in the Aethalometer-based source apportionment using radiocarbon measurements of ambient aerosol. *Atmospheric Chemistry and Physics*, *17*(6), 4229-4249. https://doi.org/10.5194/acp-17-4229-2017
